# Implementing Large Language Models in Health Care: Clinician-Focused Review With Interactive Guideline

**DOI:** 10.2196/71916

**Published:** 2025-07-11

**Authors:** HongYi Li, Jun-Fen Fu, Andre Python

**Affiliations:** 1 Center for Data Science Zhejiang University Hangzhou China; 2 School of Mathematical Sciences Zhejiang University Hangzhou China; 3 School of Medicine Children’s Hospital of Zhejiang University Hangzhou China; 4 National Clinical Research Center for Child Health Hangzhou China; 5 National Regional Center for Children’s Health Hangzhou China; 6 School of Medicine Zhejiang University Hangzhou China; 7 Centre for Human Genetics Nuffield Department of Medicine University of Oxford Oxford United Kingdom

**Keywords:** large language model, LLM, clinical, artificial intelligence, AI, digital health, LLM review

## Abstract

**Background:**

Large language models (LLMs) can generate outputs understandable by humans, such as answers to medical questions and radiology reports. With the rapid development of LLMs, clinicians face a growing challenge in determining the most suitable algorithms to support their work.

**Objective:**

We aimed to provide clinicians and other health care practitioners with systematic guidance in selecting an LLM that is relevant and appropriate to their needs and facilitate the integration process of LLMs in health care.

**Methods:**

We conducted a literature search of full-text publications in English on clinical applications of LLMs published between January 1, 2022, and March 31, 2025, on PubMed, ScienceDirect, Scopus, and IEEE Xplore. We excluded papers from journals below a set citation threshold, as well as papers that did not focus on LLMs, were not research based, or did not involve clinical applications. We also conducted a literature search on arXiv within the same investigated period and included papers on the clinical applications of innovative multimodal LLMs. This led to a total of 270 studies.

**Results:**

We collected 330 LLMs and recorded their application frequency in clinical tasks and frequency of best performance in their context. On the basis of a 5-stage clinical workflow, we found that stages 2, 3, and 4 are key stages in the clinical workflow, involving numerous clinical subtasks and LLMs. However, the diversity of LLMs that may perform optimally in each context remains limited. GPT-3.5 and GPT-4 were the most versatile models in the 5-stage clinical workflow, applied to 52% (29/56) and 71% (40/56) of the clinical subtasks, respectively, and they performed best in 29% (16/56) and 54% (30/56) of the clinical subtasks, respectively. General-purpose LLMs may not perform well in specialized areas as they often require lightweight prompt engineering methods or fine-tuning techniques based on specific datasets to improve model performance. Most LLMs with multimodal abilities are closed-source models and, therefore, lack of transparency, model customization, and fine-tuning for specific clinical tasks and may also pose challenges regarding data protection and privacy, which are common requirements in clinical settings.

**Conclusions:**

In this review, we found that LLMs may help clinicians in a variety of clinical tasks. However, we did not find evidence of generalist clinical LLMs successfully applicable to a wide range of clinical tasks. Therefore, their clinical deployment remains challenging. On the basis of this review, we propose an interactive online guideline for clinicians to select suitable LLMs by clinical task. With a clinical perspective and free of unnecessary technical jargon, this guideline may be used as a reference to successfully apply LLMs in clinical settings.

## Introduction

### Background

Large language models (LLMs) play a growing role in both medical research and clinical practice, fundamentally reshaping health care approaches [1-7]. LLMs are transformer-based (see the glossary in Multimedia Appendix 1 [1,7-59]) models trained using self-supervised learning (see the glossary in Multimedia Appendix 1) on very large amounts of textual data from various sources, including the internet, books, or articles. These models may learn complex word relationships and potential language use patterns from text data to produce natural language output that is indistinguishable from that of humans [8]. LLMs are capable of performing various tasks in the medical domain, such as case report generation [60] and medical question answering [4]. The idea of using LLMs in medicine started to emerge after the release of ChatGPT by OpenAI in November 2022. ChatGPT [9] is an LLM-based chatbot into which users can feed a “prompt” (see the glossary in Multimedia Appendix 1) in the form of natural language or a series of iterative prompts instructing it to produce a specific output, which mimics human conversation. Since its introduction, major technology giants have joined the competition by proposing alternative LLMs, such as Google’s Bard, which was replaced by Gemini [61], and LaMDA [62]. ChatGPT is built from a fast evolution of LLMs that started in 2018 with GPT-1 [63], which used approximately 7000 unpublished books and approximately 1 billion words from additional datasets for pretraining (see the glossary in Multimedia Appendix 1). In 2019, GPT-2 [64] increased its capability with 1.5 billion parameters and used 40 GB—1 GB contains approximately 166 million words from text data for pretraining. It was only in 2020, with the third generative pretrained transformer version, GPT-3 [65], that it reached humanlike accuracy in tasks such as question answering, advanced search, and language translation. One key development in GPT-3 included few-shot [19] and zero-shot (see the glossary in Multimedia Appendix 1) reasoning capabilities in some cases, along with a considerable increase in the size of the parameters, with 175 billion parameters and a pretraining dataset of approximately 45 TB of text. Eventually, the reinforcement learning (see the glossary in Multimedia Appendix 1) from human feedback method was applied in the fine-tuning (see the glossary in Multimedia Appendix 1) process in GPT-3.5, also known as ChatGPT [9]. Reinforcement learning from human feedback trains a reward model by collecting human ranking feedback on the model outputs, which can simulate human evaluation and human reward of the quality of the generated text. The LLM is then automatically fine-tuned and optimized through iterative algorithms to align the output of the language model with human preferences. This approach may reduce toxic output such as text with hateful content and make the output form more human-friendly [9,30].

Models that consider only 1 type of input data are unlikely to satisfy all requirements from clinicians, who often need to make decisions based on multiple information sources. Led by GPT-4, LLMs that can accept multiple types of input data, which are called multimodal LLMs (MLLMs; see the glossary in Multimedia Appendix 1), have progressively filled the gap. GPT-4 is a general-purpose MLLM that accepts images and text as input and produces text as output, reaching human levels on a variety of professional and academic benchmarks [66]. A more recent version, GPT-4o (along with a more affordable version named GPT-4o mini), offers multimodal interactions while retaining the powerful language comprehension capabilities of GPT-4, enabling any combination of text, image, and audio input and output. MLLMs have been applied in radiology and pathology, including applications such as rare disease diagnosis [67], radiology report generation [68], and pathology image searching and classification [69]. Attempts to combine genomic data with text have led to the analysis of gene-phenotype relationships and facilitated genetic discovery [70].

### Reviews of LLMs in Medicine

Systematic reviews of LLMs have highlighted their strengths, limitations, and future development directions in health care [10,11,71,72]. Reviews focused on specialized applications of LLMs have covered radiation oncology [73], cardiology [74], gastroenterology [75], oral and maxillofacial surgery [76], clinical laboratory medicine [77], and psychology [78]. Tian et al [12] investigated the performance of LLMs in biomedicine from the perspective of traditional natural language processing (see the glossary in Multimedia Appendix 1) tasks such as information extraction and question answering. Chang et al [13] offered a comprehensive review of LLM evaluation methods, putting emphasis on 3 key dimensions: what to evaluate, where to evaluate, and how to evaluate. Wornow et al [79] examined and created a taxonomy for 84 foundation models (see the glossary in Multimedia Appendix 1) trained on nonimaging electronic medical record data. Hu et al [80] extended the scope of previous reviews by investigating applications of MLLMs in medical imaging, which essentially focused on bidirectional encoder representations from transformers (BERT)–based models, long short-term memory, and ChatGPT. Li et al [81] drew development and deployment road maps of artificial general intelligence (AGI; see the glossary in Multimedia Appendix 1) models (mainly MLLMs) in medical imaging while also providing key insights into potential challenges and pitfalls. While there is no consensus on what AGI is, one may view an AGI system as a form of artificial intelligence (AI) with a general scope with the ability to perform well across various goals and contexts [17]. Finally, Yuan et al [82] provided a broad review of the applications and implications of LLMs in medicine, especially MLLMs, and discussed the emerging development of LLM-powered autonomous agents.

So far, the literature has not provided a systematic guideline for clinicians and other practitioners in health care to select LLMs relevant and suitable to their needs. Addressing this gap is crucial to ensure that clinicians can, without previous specific expertise, obtain sufficient information to envisage the deployment of user-friendly LLMs that are truly beneficial in real-world clinical settings. In this study, we systematically examined the role that LLMs have played in the completion of clinical tasks within a patient-oriented clinical workflow (Figure S1 in Multimedia Appendix 1). We complemented this review with an interactive online guideline that offers guidance to clinicians to select LLMs that are suitable to accomplish their tasks based on their answers to a series of questions.

## Methods

### Search Strategy

The search strategy was designed to identify relevant studies that cover the most comprehensive sets of available data. We used a keyword-combination search strategy that allowed us to conduct an extensive search in IEEE Xplore, PubMed, ScienceDirect, and Scopus. Considering the timing of the recent emergence of MLLMs, we also conducted searches in arXiv, including the latest non–peer-reviewed studies. The keywords used included generic keywords (ie, *large language models* and *LLMs*) to conduct an extensive search and specific keywords (specific LLM names, eg, *GPT* and *LLaMA*) to conduct an enhanced search to minimize omissions. The keyword search identified 15,699 potentially relevant articles. After excluding duplicates, 10,768 articles remained (n=917, 8.52% from arXiv and n=9851, 91.48% from the other databases). We collected articles from 2022, which coincides with the year in which LLMs became applicable in clinical settings—ChatGPT was initially released in November 2022 [9]. For different databases, the search keywords were, in principle, identical, but the search strategy may change slightly (Table 1 and Tables S1-S5 in Multimedia Appendix 1).

**Table 1:** Generic keyword search strategy per source—list of the search strategies used by source of academic research to select relevant studies that used large language models in medical (including clinical) applications.

Source	Search strategy
PubMed	(“LLMs”[Title/Abstract] OR “large language models”[Title/Abstract]) AND ((medic*) OR (clinical) OR (health*))^a^
ScienceDirect	Title, abstract, and keywords: “(LLMs) AND ((medical) OR (clinical) OR (healthcare) OR (medicine))”^b^ Title, abstract, and keywords: “(large language models) AND ((medical) OR (clinical) OR (healthcare) OR (medicine))”^b^
Scopus	(TITLE-ABS-KEY (“large language models” OR “LLMs”) AND TITLE-ABS-KEY (“clinical” OR “medic*” OR “health*”)) AND (LOAD-DATE >20220101) AND (LOAD-DATE <20250331) AND (LIMIT-TO (DOCTYPE, “ar”)) AND (LIMIT-TO (LANGUAGE, “English”))
IEEE Xplore	((“Full Text .AND. Metadata”: “large language models” ) OR (“Full Text .AND. Metadata”: “LLMs” )) AND ((“Abstract”: medic*) OR (“Abstract”: clinical) OR (“Abstract”: health*))^c^
arXiv	AND abstract=“large language models” OR LLMs; AND abstract=medic* OR health* OR clinical; AND abstract=multimodal^d^

^a^Time span: January 1, 2022, to March 31, 2025.

^b^Time span: January 1, 2022, to March 31, 2025; article type: research articles.

^c^Time span: January 1, 2022, to March 31, 2025; paper type: journals.

^d^Date range from January 1, 2022, to March 31, 2025.

### Defining the Inclusion and Exclusion Criteria

We then defined inclusion and exclusion criteria first by limiting the scope of the journals to which the articles belonged. Specifically, we used the source publication search function in the Scopus database; set the subject area to *biochemistry, genetics and molecular biology*, *computer science*, *dentistry*, *health professions*, *medicine*, *neuroscience*, and *nursing*; and set the minimum number of citations (counts for a 4-year time frame) of the journal to 13,000, which restricted the search to 509 journals. We believe that focusing on highly cited journals helped identify those that published the most relevant and influential research articles on the clinical application of LLMs. We conducted screening of the articles searched in IEEE Xplore, PubMed, ScienceDirect, and Scopus and retained 18.52% (1824/9851) of the screened articles published in the 509 journals. Of these 1824 retained articles, we further excluded 735 (40.3%) that were not focused on LLMs, 275 (15.08%) nonresearch articles (eg, reviews and commentaries), and 630 (34.54%) articles without clinical applications of LLMs, yielding a total of 184 relevant articles. For arXiv, we focused on identifying articles with innovative MLLM approaches by screening abstracts that contained clinical applications of LLMs, which led to 9.4% (86/917) of relevant articles among those screened. Combining the results of the aforementioned 2 parts, we retained a total of 270 articles in our review. The literature search and screening process were independently performed by HL and independently reviewed by AP. However, we acknowledge that, due to the rapid development of LLMs for clinical applications, it remains challenging to systematically identify all relevant studies. In addition, publication bias may influence the existing literature in favor of reporting positive findings. Of the studies we included, only 8.9% (24/270) reported negative results for the clinical use of LLMs, which could affect the overall perception of their clinical effectiveness.

We manually extracted the required data from the 270 studies included in our review. Extracted information included clinical tasks and subtasks, LLMs used in each study, and the best-performing LLMs reported by the original authors. If a study reported negative or underperforming results for LLMs in clinical use, those were not recorded as the best-performing models. In addition, we gathered detailed information about each model, including model size (see the glossary in Multimedia Appendix 1), resource consumption, and accessibility (see the PRISMA [Preferred Reporting Items for Systematic Reviews and Meta-Analyses] checklist in Multimedia Appendix 2). Data extraction was independently performed by HL and independently reviewed by AP.

**Figure 1:**
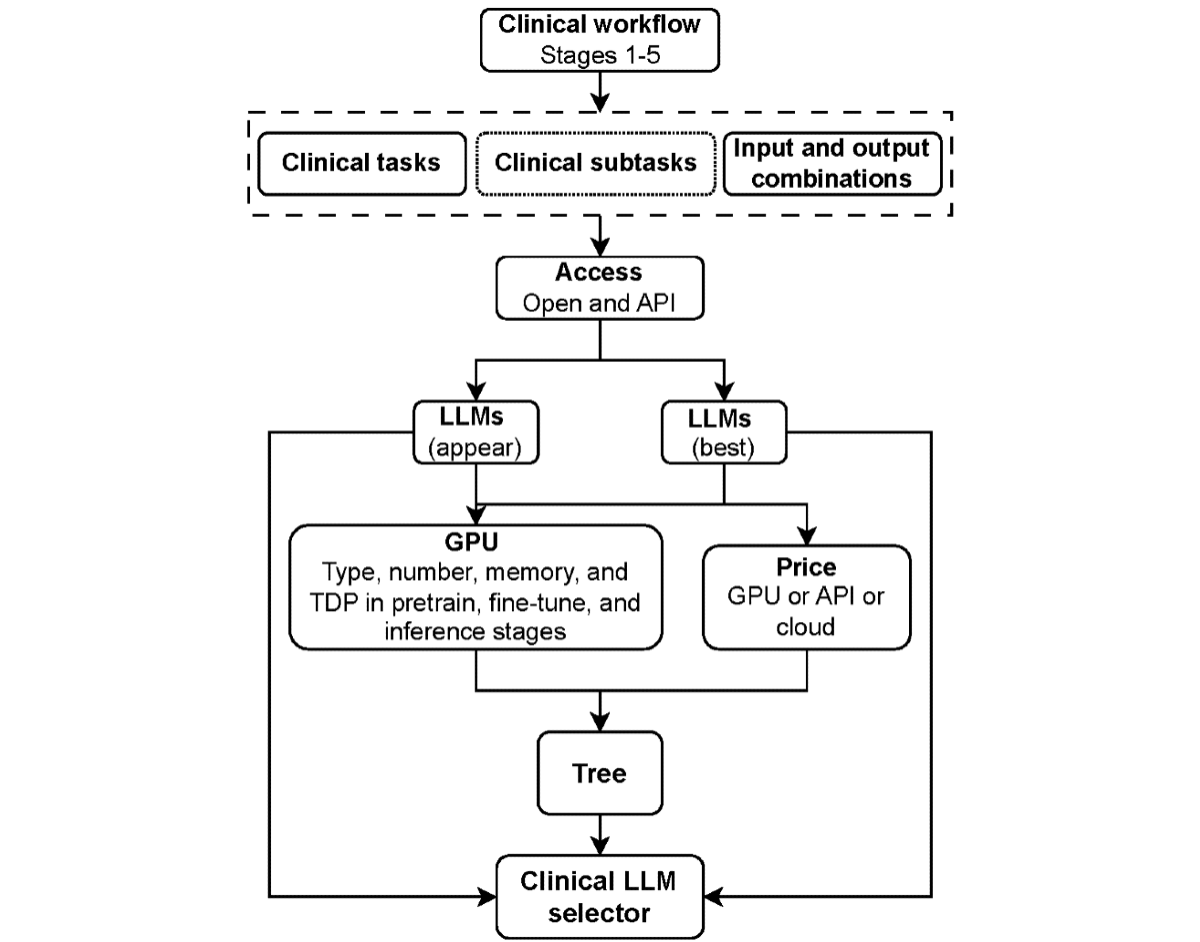
Clinical large language model (LLM) selector—schematic description of the clinical LLM selector tree for clinicians to select LLMs suitable to their needs. API: application programming interface; GPU: graphics processing unit; TDP: thermal design power.

To help clinicians and health practitioners select LLMs, we proposed an interactive guideline with a clinical LLM selector tool that relies on a large-scale decision tree containing hundreds of nodes (general description in Figure 1). Using LLM names as keys, we recorded the number of appearances of 330 identified LLMs and their frequency of performing best by clinical task and input and output modalities. We then used input modality, output modality, and clinical task category as branch nodes for tree classification. Clinical tasks and subtasks were merged when possible (eg, the treatment plan recommendation generation and clinical letter generation subtasks of stage 3 would be merged into a text generation task). We used the access methods of LLMs as nodes but excluded access-restricted LLMs (see the glossary in Multimedia Appendix 1) to recommend LLMs that can be obtained and used by all. We also recorded the resource consumption of the LLMs from the corresponding paper and graphics processing unit (GPU) specificities used in the pretraining, fine-tuning, or inference (see the glossary in Multimedia Appendix 1) phases (fixed costs), as well as the purchase price of the GPUs, the price of the token for application programming interface (see the glossary in Multimedia Appendix 1) access, and the price of the GPUs for the use of a cloud service (which is regularly updated in our interactive guideline). Thus, we merged LLM information to a categorical binary tree where the branch nodes correspond to specific questions to be answered and the leaf nodes are the specific information on the LLMs that satisfy the conditions.

## Results

### Gathering Information on LLMs Associated With Clinical Tasks to Provide Practical Guidance

**Figure 2:**
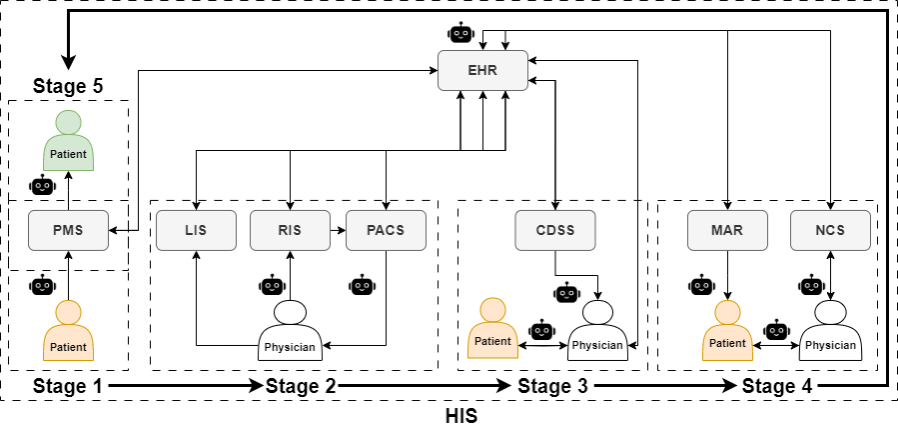
Integration of large language models (LLMs) in a hospital information system (HIS) in a 5-stage clinical workflow. HIS subsystem modules interact with 5 stages of a typical clinical workflow: registration and department guidance (stage 1), prediagnosis and examination (stage 2), diagnosis and treatment planning (stage 3), treatment and hospitalization (stage 4), and discharge and follow-up (stage 5). Patients registered are shown in orange and are shown in green when discharged. CDSS: clinical decision support system; EHR: electronic health record; LIS: laboratory information system; MAR: medication administration record; NCS: nurse call system; PACS: picture archiving and communication system; PMS: practice management system; RIS: radiology information system; robot symbol: possible clinical applications of LLMs (or multimodal LLMs).

To provide practical guidance for clinicians to select LLMs (the interactive guideline), we considered the integration of LLMs into a hospital information system within the 5-stage clinical workflow (Figure 2).

This allowed us to link applications of LLMs from our review with clinical tasks associated with the path of a typical patient in a hospital. In stage 1, patients register their personal information through a practice management system and make appointments with different departments [83], which is synchronized with an electronic health record (EHR) database. In stage 2, a physician arranges examinations, with possible laboratory results sent from the laboratory information system to the EHR database. If radiology images are generated, they are stored in the picture archiving and communication system [84] and synchronized along with radiology reports to the EHR database. Stage 3 includes diagnosis of the disease and treatment planning recommendations based on data from the EHR system. In stage 4, the medication administration record system accepts medication orders, links with the EHR to track medication administration, updates medication records, and ensures consistency with the treatment plan. A nurse call system provides help to patients (eg, daily monitoring data and nursing) synchronized with the EHR database. In stage 5, discharge summaries and all clinical data for the patient are recorded in the EHR system, and treatment billing and follow-up appointments are completed in the practice management system [83] and synchronized to the EHR system.

### LLMs May Assist but Not Replace Humans in Clinical Tasks

**Figure 3:**
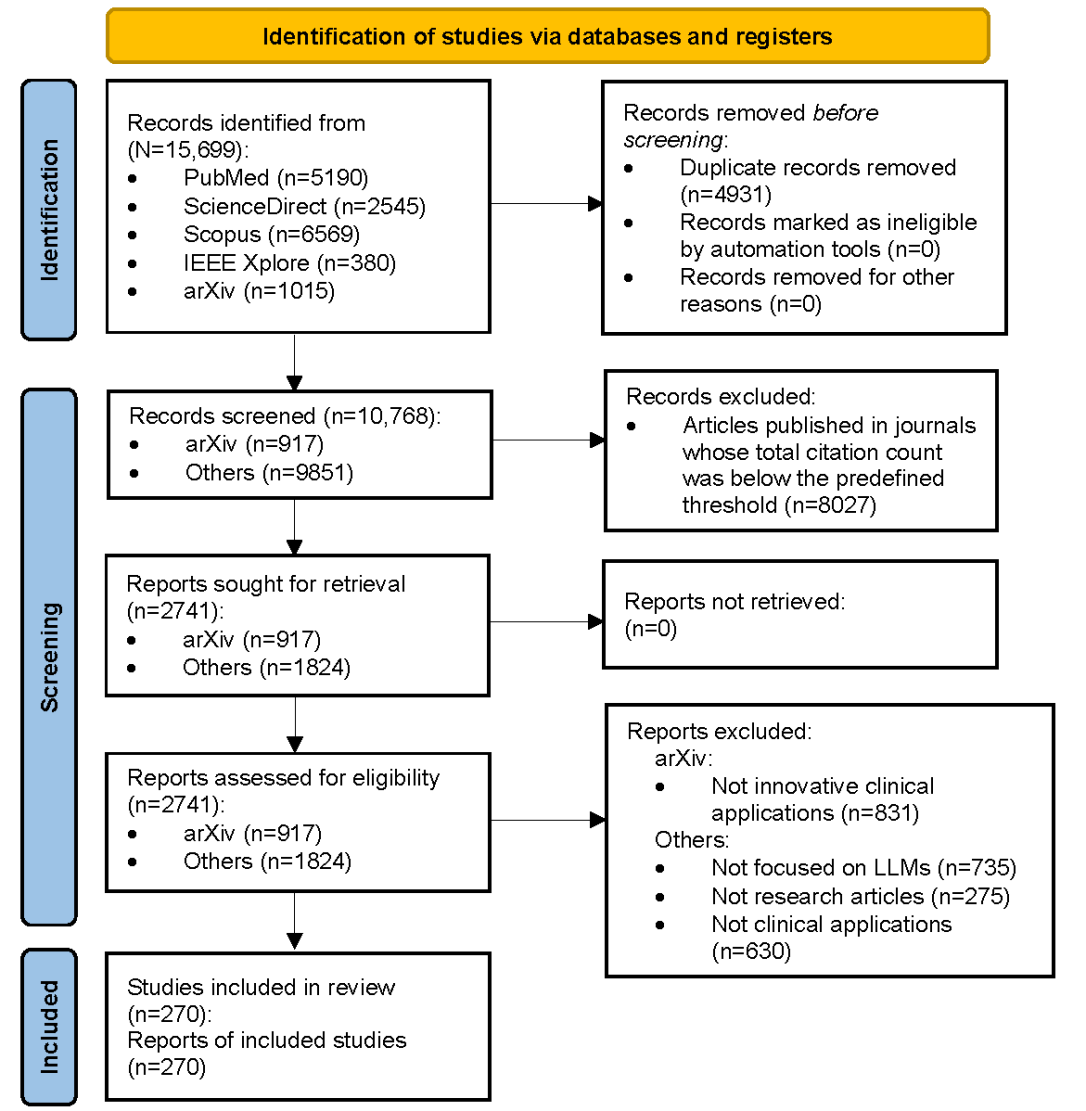
Literature review screening process summary. The literature review process aimed at identifying articles that were relevant to large language models (LLMs) used in clinical work. A total of 15,699 articles were obtained using a keyword-combination search strategy. Following a selection process putting emphasis on the selection of innovative clinical applications of LLMs, we kept a total of 270 papers.

**Figure 4:**
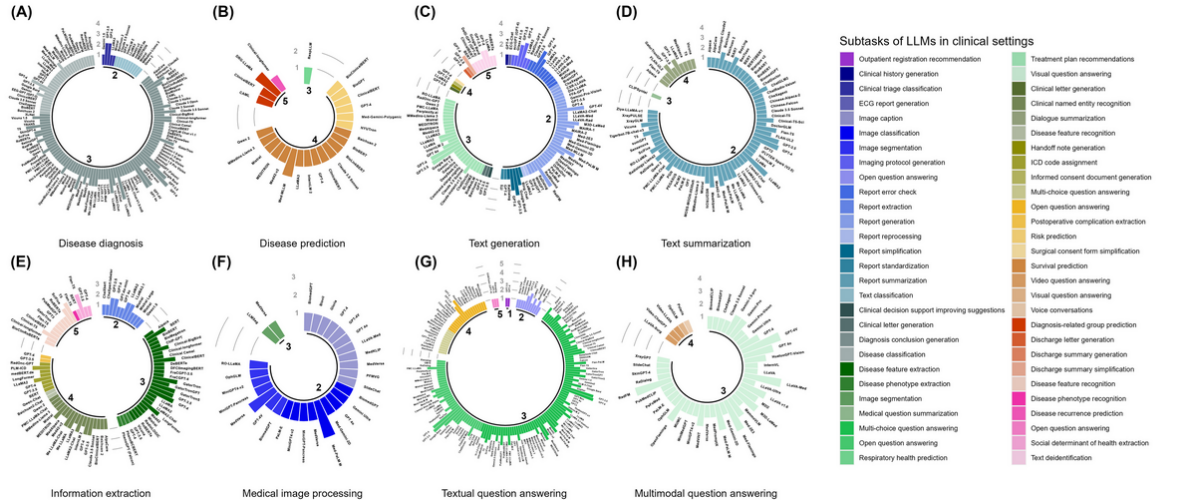
Log-transformed frequency of large language models (LLMs) by clinical stage, task (A-H), and subtask. Each panel shows the log-transformed number of studies using each LLM for specific subtasks within a 5-stage clinical workflow. Bar height reflects study count per LLM per subtask and stage. Colors indicate 56 subtask categories across stages. For example, panel F (medical image processing) spans stages 2 and 3, where stage 3 includes the image segmentation subtask for which MedVersa and LLMSeg were used in 1 study. ECG: electrocardiography; ICD: International Classification of Diseases.

We gathered 330 LLMs that appeared in the literature and recorded the number of times each model was applied in subtask categories, as well as the number of times it performed best in its context (see details in the Methods section and the PRISMA flow diagram in Figure 3). We only reported the best-performing models as stated in the original papers and did not quantitatively compare models from different studies because the validation sets and evaluation metrics used in each study may vary. We found that LLMs were used in assisting clinicians in a large variety of tasks (Figure 4), such as diagnosis of diseases, answering medical questions, and assigning *International Classification of Diseases* codes to patients, mainly in clinical stages 2 to 5. Surprisingly, of the 270 studies, we found only 1 (0.4%) in stage 1 on tasks in which LLMs could be used to optimize the patient visit process, such as guiding and assisting patients in registering for hospital admission or guiding patients to transfer between complex departments. This gap may stem from the need to develop specialized health care agents (ie, software systems and applications that can assist with specific tasks in the health care environment [85]). Stages 2, 3, and 4 involve 6 (or more) clinical tasks, among which stage 3 involved all clinical tasks and the largest number of LLMs, whereas stage 2 involved the largest number of subtask categories. These 3 stages are indeed key stages in which LLMs were used to assist physicians in completing examinations and making diagnoses, as well as to provide assistance in the treatment process. Figure 4 shows that all 3 tasks—text generation, information extraction, and textual question answering—can be applied to 4 (or more) clinical stages, among which Textual question answering is applied in all clinical stages, which may be related to the generalizability of the tasks themselves. The variety of LLMs used in the disease prediction and medical image processing tasks was lower than that for other tasks, which suggests that these tasks require domain-specific algorithms. Traditional machine learning methods may offer a suitable alternative for disease prediction [86]. The number of LLMs used in the multimodal question answering task was much lower than the number of LLMs used in the textual question answering task, indicating that the development of MLLMs is still in the initial stage and has great potential. GPT-3.5 and GPT-4, which are both easily accessible with little additional resource consumption, were omnipresent, with applications in almost all clinical tasks. BERT, Llama 2, Flan-T5, MedAlpaca, and ClinicalBERT were also used frequently and had the advantage of being open-source LLMs (see the glossary in Multimedia Appendix 1), which facilitates their development.

### A Few Versatile LLMs Tend to Perform Best Overall, but Performance Remains Context Specific

**Figure 5:**
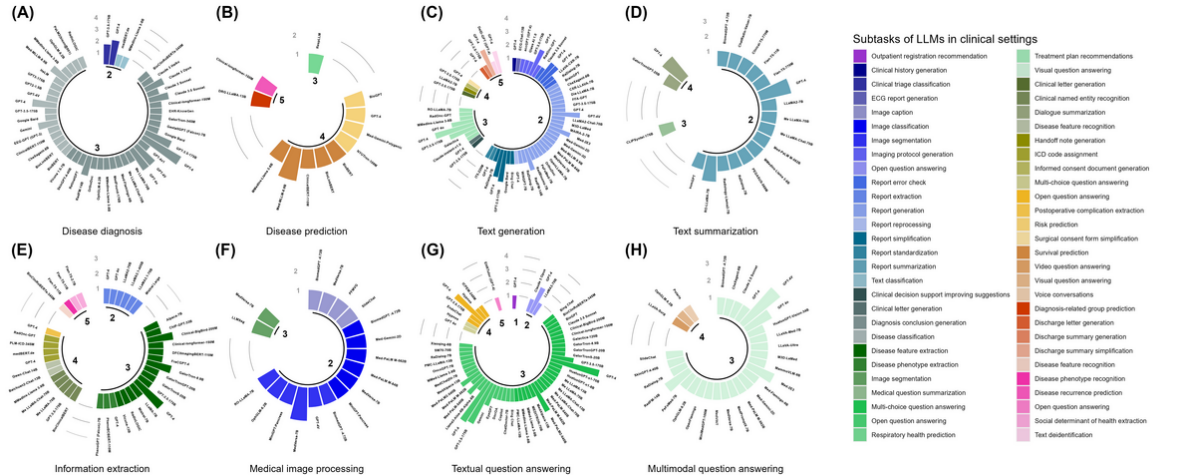
Best-performing large language models (LLMs) by clinical stage, task (A-H), and subtask. Each panel shows the log-transformed frequency of LLMs that performed best for specific subtasks within a 5-stage clinical workflow. Bar height indicates how often an LLM performed best for each subtask, under a given stage. Colors denote 56 subtask categories across stages. For example, panel F (medical image processing) covers stages 2 and 3, with stage 3 including the image segmentation subtask where MedVersa and LLMSeg each ranked best in 1 study. ECG: electrocardiography; ICD: International Classification of Diseases.

The variety of LLMs that showed the best performance in a given context remains within a limited circle (Figure 5). In Figure 5, we annotated model size to the LLM version to facilitate the identification of the exact specificities of the best-performing model. For example, the Llama 2 model may exhibit various levels of predictive performance with 7, 13, and 70 billion parameters. Therefore, it was essential to distinguish them based on model size. Some subtasks, such as report generation (stage 2), Diagnosis conclusion generation (stage 3), and multi-choice question answering (stage 3), were highly competitive, including dozens of different best-performing LLMs. In contrast, subtasks such as ECG report generation (stage 2), video question answering (stage 4), and discharge summaries generation (stage 5) only had 1 best-performing LLM, which may reflect the difficulty and popularity of the subtasks. GPT-3.5, GPT-4, GPT-4V, GPT-4o, Llama 2, and Llama 3 were commonly used in disease diagnosis, among which GPT-4 exhibited the best performance the most times. Med-MLLM and DRG-LLaMA were often used to predict diseases, with Med-MLLM-8.9B usually outperforming its competitors. The generation of text was usually conducted via GPT-3.5 and GPT-4, which were similarly the best-performing models. For text summarization, while Llama 2 was the most frequently used, GPT-4 and RoLlama-7B performed best more frequently. GPT-3.5, GPT-4, Flan-T5, GatorTron, BERT, and Llama 2 were widely used to extract information, but GPT-4, Clinical-BigBird-200M, and GatorTron-8.9B often performed best. Processing medical images was most commonly performed using Med-PaLM M and MedVersa, with MedVersa-7B showing the best performance overall. For textual question answering, GPT-3.5 or GPT-4 remained the main choice for most users, showing the best overall performance. The most commonly used models for answering multimodal questions were GPT-4V and LLaVA-Med, and GPT-4V was still the model with the best performance the most times. Overall, GPT-3.5 and GPT-4 were the most versatile, with applications in approximately 30 subtasks (Figure 5).

However, these versatile LLMs may not always perform well, especially without fine-tuning and when applied in specialized and complex tasks such as disease information extraction, medical image processing, and treatment recommendations. In a clinical context, GPT-3.5 failed to provide treatment plans that would align with the quality and credibility that experts may aim for [87]. In some cases, GPT-4 also showed some difficulties in extracting relevant clinical information from EHRs [88,89]. GPT-4V was often applied to identify and describe possible diseases based on general medical knowledge rather than extracting relevant information from medical images for diagnosis purposes [68]. GPT-4V was also unable to reliably interpret radiological imaging studies and tended to ignore images, fabricate results, and misidentify details [90]. To improve LLMs’ performance in related tasks, a lightweight fine-tuning method is prompt engineering (see the glossary in Multimedia Appendix 1), which aims to guide the model to produce outputs that better meet the task requirements by designing well-intended prompts with clear structure. The importance of effective prompt engineering in ensuring the accuracy of model-generated suggestions and improving clinical efficiency has also been emphasized [91]. Therefore, the use of validated prompt templates [14,92] can be considered to improve model performance. When prompt engineering also fails to meet the task requirements, fine-tuning using professional medical data can inject specific domain knowledge into the model, reduce dependence on large-scale data, and improve model parameter efficiency. Smaller, more efficient parametric LLMs with pretraining on clinical text can match or outperform larger LLMs trained on general text [93] while reducing the computational resources (see the glossary in Multimedia Appendix 1) required to support the operation of large-scale LLMs.

### Closed-Source LLMs May Lead the Way, but It Comes With a Price

**Figure 6:**
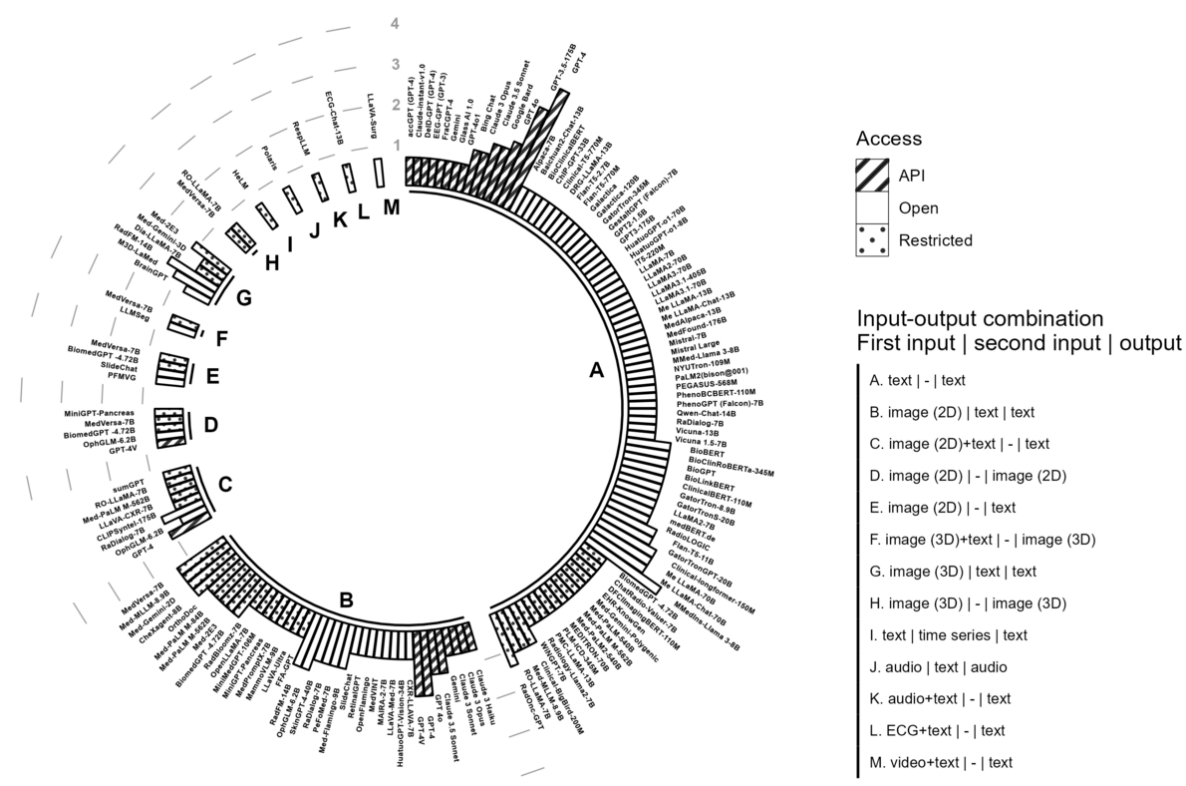
Summary of input-output combinations and access paths for best-performing large language models (LLMs). Panels A to M show log-transformed counts of subtasks for which each LLM (with model size annotated) performed best under specific input-output combinations based on original study data. Inputs are grouped as mandatory (first) and optional (second); the dash (–) denotes absence of optional input. Bar height reflects the (log) count of subtasks for which an LLM performed best, and fill patterns indicate access paths—application programming interface (API; hachures), restricted LLMs (dots), and open-source LLMs (no filling). ECG: electrocardiography.

**Figure 7:**
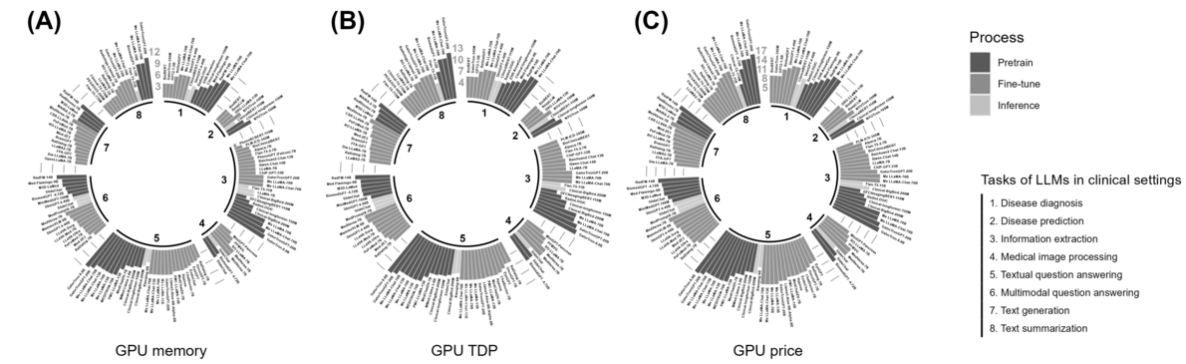
Computational resources and costs of the best-performing large language models (LLMs). Panels A to C show graphics processing unit (GPU) memory, thermal design power (TDP), and price (log-transformed in gray) for each LLM (annotated on the bars) based on literature sources. GPU specifications are from NVIDIA documentation; the missing memory for A100 or A800 is assumed as 40 GB. For GPUs that had both PCIe and SXM architectures, we assumed the use of PCIe with fluctuations in the TDP data. Prices are from eBay (April 2024) and for reference only. For LLMs using multiple GPUs, memory, TDP, and cost are summed. The bar color is associated with the pretraining (dark gray), fine-tuning (gray), and inference (light gray) stages in which the GPU is activated. Numbers 1 to 8 represent clinical task categories.

A large number of LLMs appeared to perform best in unimodal settings (Figures 6 and 7), which suggests that a large amount of literature has focused on applying LLMs to unimodal data and the performance of LLMs may be context specific. Although the clinical tasks with the largest number of best-performing LLMs were those with text-only modalities, the LLMs were increasingly capable of handling multimodal inputs beyond text, enabling more diverse clinical applications. Some LLMs such as RoLlama, RadFM, MedVersa, and Med-2E3 can process both 2D and 3D images and textual data in, for example, diagnosis and treatment planning, which often require the analysis of a combination of radiological images (x-ray, computed tomography [CT], and magnetic resonance imaging). LLMs such as Polaris (panel J in Figure 6) can use and generate audio files, which may offer a potential substitute to, for example, exchanges between patients and caregivers during hospitalization. Similarly, RespLLM (panel K in Figure 6) incorporates both text and audio data to generate diagnoses associated with respiratory health. Moreover, ECG-Chat (panel L in Figure 6) can use electrocardiography (ECG) signaling data to generate ECG medical reports by aligning ECG data features with textual data at a fine-grained level. The open-source video dialog model LLaVA-Surg (panel M in Figure 6) gained the ability to answer open-ended questions about surgical videos from a fine-tuning procedure carried out on a large-scale surgical video instruction-tuning dataset.

The type of access to LLMs also influences the choice of medical professionals (application programming interface, restricted LLMs, and open-source LLMs; see the bars in Figure 6). While open-source LLMs may offer a larger variety of model choices in the textual modality, our review suggests that closed-source LLMs (see the glossary in Multimedia Appendix 1) such as GPT-4, GPT-4V, and MedVersa tend to perform better overall, especially in the presence of oligopolies in specific modalities (eg, input-output combinations in panels B and H-L in Figure 6). This may be explained by the large amount of resources required to acquire high-quality multimodal medical data and training processes. To provide decision makers in medical institutions with a comparative basis for the resource consumption of LLM training and deployment, we examined the information on GPU resources required by the LLMs that performed best in different clinical tasks during pretraining, fine-tuning, and inference (this information only represents the resource consumption reported in the literature and may not necessarily coincide with the minimum computational resource requirements to use the LLMs). Figure 7 shows memory, thermal design power, and reference price by LLM. The GPU resource requirements of the LLMs tended to increase with their model size and the size of the training set during the pretraining stage. While resource requirements to train LLMs are likely to gradually decrease [1], cost-effective LLMs may be favored by institutions with a restricted budget.

### A Promising Future for Generalist LLMs for Centralized Multitasking in a Clinical Setting

**Figure 8:**
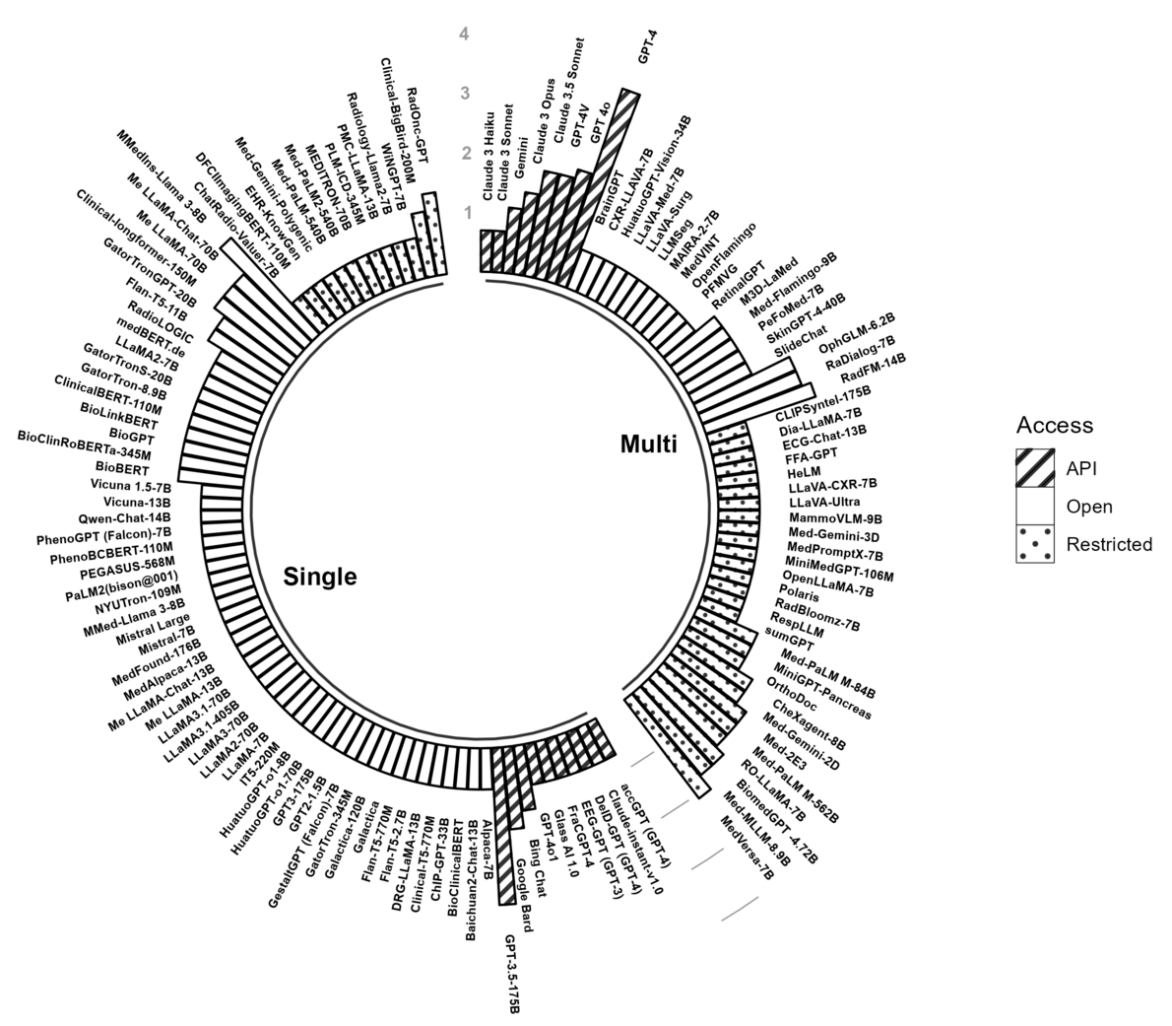
Modality and access paths of the best-performing large language models (LLMs). Bars show the log-transformed number of subtasks for which each LLM (with name and model size labeled) performed best categorized by single-modal (Single) and multimodal (Multi) input and output based on a literature review. We excluded LLMs that did not perform best in any subtask. Bar patterns represent application programming interface (API; stripes), restricted LLMs (dotted area), and open-source LLMs (blank).

One might wish for a generalist clinical LLM acting as an intelligent hub, as envisioned by Moor et al [15] in their concept of generalist medical AI. Such LLMs do not require model parameter updates to perform various clinical tasks but might be required to be trained or fine-tuned using specialized clinical medical knowledge. Figure 8 shows that, of the noncommercial LLMs, MMedIns-Llama 3-8B performed best among the single-modal clinical LLMs the most times. Clinical-Longformer-150M also performed well in a large number of clinical subtasks, with the advantages of a relatively small model size (<1 billion parameters) and being an open-source LLM. RaDialog 7B [94], Med-PaLM M (84B and 562B) [95], RadFM 14B [96], and MedVersa 7B [97] seem promising generalist clinical LLMs (here, we exclude commercially available LLMs [eg, GPT-3.5 and GPT-4], which may perform well but might not be suitable in most clinical settings to comply with medical data privacy policies). RaDialog is an open-source LLM specifically designed for radiology tasks based on x-ray images, such as processing radiology reports and interactive question answering. Med-PaLM M can be applied to a wider range of tasks, including processing radiology reports, medical question answering, and medical image classification. It can use medical images from multiple sources (eg, radiology, pathology, and dermatology) as inputs, as well as genomic data. However, the scope of its tasks remains limited. It lacks the ability to carry out core tasks related to disease diagnosis and treatment. Moreover, it is a restricted LLM and requires 84 billion parameters to perform well. Its largest model version is composed of 562 billion parameters, which requires relatively large computational resources and energy, making it difficult to be deployed in ordinary medical institutions. The open-source LLM RadFM supports both 2D and 3D images (eg, CT, magnetic resonance imaging, x-ray, and ultrasound) from 17 source types such as chest or breast scans and can perform disease diagnosis, report processing, and medical visual question answering tasks. It is also limited to the field of radiology, and its ability regarding the specialized clinical medical knowledge required for clinical tasks has yet to be confirmed. MedVersa [97] is a restricted generalist learner that enables flexible learning and tasking for medical image interpretation (x-ray, CT, and dermatology images). It can incorporate a wide range of inputs, including images of various types (eg, 2D and 3D, frontal and lateral, or multiperiod images) and natural language requests. However, it only focuses on vision-language or vision-centric tasks related to image interpretation without considering disease diagnosis and treatment plan development.

### Potential and Pitfalls of the Integration of LLMs Into a Clinical Workflow

LLMs and MLLMs may improve the efficiency, accuracy, and quality of care in all stages of a clinical workflow (see stages 1-5 in Figure S1 in Multimedia Appendix 1). In stage 1, LLMs can help patients of all ages and walks of life register their personal information by providing clear and understandable explanations and suggest departmental appointments by taking into account patients’ descriptions of their symptoms [85], and MLLMs can provide voice support to enhance the patient experience. In stage 2, MLLMs can support radiologists in reviewing imaging results, such as pathology or radiology visual question answering [98], and can assist radiologists in generating and reviewing radiology reports [67,99]. In stage 3, MLLMs can independently make diagnoses [67,100] in parallel with clinicians, alerting them to consider or reject the MLLMs’ diagnostic results only in the event of a discrepancy, which prevents excessive workload in validating model conclusions and supports physicians in mitigating the risks inherent in clinical decision-making [101]. MLLMs can also provide personalized treatment recommendations [102] for patients based on clinical guidelines for physicians’ reference. In stage 4, MLLMs can provide ancillary nursing support for patient treatment and hospitalization [103], such as following up with patients on current medication and treatment status and answering patient questions in plain language, aiming to mimic human care and psychological guidance [104]. In stage 5, LLMs can help physicians complete discharge summaries [105] and also provide patients with detailed and complete rehabilitation recommendations and precautions.

However, the clinical integration of state-of-the-art LLMs and MLLMs remains challenging. So far, the accuracy and reliability of LLMs in making decisions for different demographics or medical conditions [1] and their compliance with data privacy–related regulations remain difficult to assess. Furthermore, LLMs may not be fully compatible with existing health care interoperability standards (eg, Health Level Seven and Digital Imaging and Communications in Medicine) [106]. Each system may require additional customizations or interfaces to accommodate the specific data input and output needs of the LLMs to ensure smooth data exchange. The ability of LLMs to make real-time decisions relies on the capability to rapidly access and process large amounts of structured or unstructured data within the EHR system, which ultimately relies on the technological advancement of hospital infrastructures.

## Discussion

### Principal Findings

While LLMs offer promising solutions in clinical settings, several limitations may prevent a broader deployment. First, many LLMs that claim to be trained on clinical texts are usually trained on a few publicly available electronic medical record datasets (eg, Medical Information Mart for Intensive Care–Chest X-Ray, Medical Information Mart for Intensive Care–III, or PubMed) or on restricted datasets within medical institutions [79]. Typically, the volume and diversity of these data cannot match the complex and varied nature of real-world data, which often leads to unreliable model outputs. In addition, more comprehensive and larger training datasets do not guarantee that the conclusions generated by LLMs will be more or sufficiently accurate and helpful in clinical practice [87,107-109].

Second, the training data may be of poor quality and may lack expert review. The training data of GPT-3.5 are text data collected from the internet [65], which include low-quality data. Med-PaLM 2 [110], which is trained using a lower amount of but higher-quality domain data, has performed close to or exceeded the state-of-the-art level on several clinical datasets. Lehman et al [93] also show that LLMs trained on highly specialized medical domain data improve parameter efficiency. High-quality clinical data remain scarce. While EHRs can theoretically hold nearly unlimited amounts of multimodal big data, their access is often restricted [10].

Third, while LLMs can generate human-preferred, coherent, and credible language outputs, these can be fabricated or inaccurate, a phenomenon known as “hallucination” [111]. ChatGPT can incorrectly assign a “young age” to a patient based on snippets of clinical records even though no age information has been given [112]. Both GPT-3.5 and GPT-4 have shown nonnegligible citation error rates in learning health system training, fabricating nonexistent fake articles in references [113]. However, chain-of-thought prompting and self-consistency can help LLMs improve their reasoning ability to achieve self-improvement [114]. GPT-4 can detect its own hallucination errors when provided with complete conversation records [115]. Statistics-based semantic entropy [116] is a universal method for detecting illusions in LLMs even for new questions with unknown answers.

Fourth, LLMs are inherently black boxes, usually lacking transparency and interpretability, and, therefore, are often not trusted by decision makers. Although explainable AI tools such as attention score visualization [117], Shapley Additive Explanations values [118], and saliency methods [119] can provide local logic for model operation, this information may not necessarily help clinicians improve their understanding and trust in LLMs. To make informed decisions, clinicians usually require a high degree of post hoc interpretability, such as information about the referenced literature and how model outputs translate into clinical metrics [120]. Bringing in human experts for validation and review may be beneficial for clinicians to make decisions without obtaining a full explanation of the underlying AI system’s output [10].

Fifth, the clinical use of LLMs raises strong ethical concerns. Previous research has shown that, if harmful biases are present in the training data, language models may encode and perpetuate these biases [121,122]. Instead of appropriately modeling the demographic diversity of medical conditions on some clinical vignettes, GPT-4 produces differential diagnoses with stereotypes [123] and may include racial bias [11]. LLMs may be vulnerable to external attacks that jeopardize the privacy and security of training data [124], such as how GPT-4 showed vulnerability to adversarial prompt attacks [125], and medical LLMs are susceptible to deliberately implanted misinformation [126]. While training LLMs using deidentified patient data locally might appear as a safe option, most health care organizations cannot afford advanced IT technical teams and infrastructure. Instead, they might opt for a cloud-based solution to reduce maintenance and operational costs [127]. Whether LLMs are used on the premises or in the cloud, strict security measures such as differential privacy, deidentification, and federated learning must be implemented to minimize the risk of data breaches [128]. Commercial LLMs might not be accessible to institutions and researchers with limited funds, thereby potentially widening the existing digital divide [129]. While some governments have proposed regulatory efforts, such as the European Union’s AI Act [130] and Canada’s Artificial Intelligence and Data Act [131], the existing legal framework often remains inadequate and ambiguous when it comes to the deployment of LLMs in clinical settings. The lack of transparency and accountability mechanisms continue to raise public concerns about the potential risks of widespread integration of LLMs with digital health [132].

Sixth, the evaluation of the performance of LLMs in clinical practice remains difficult. Different tasks may be evaluated from different angles (eg, medical question answering tasks can be evaluated from the perspective of output accuracy, medical reasoning ability, coherence, and possibility of harm [110]). Although evaluation methods and datasets have been proposed for different evaluation dimensions [133-136], there is still no consensus on a standardized and comprehensive evaluation framework, which requires further investigation. Moreover, models trained and validated on research datasets are also difficult to deploy directly to medical institutions due to the large differences between laboratory and clinical settings. Gollub and Benson [137] highlight 4 key differences between experimental and real clinical data: standardization of access, quality of the data, the presence of a control group, and reporting methodology (quantitative or qualitative). Therefore, to evaluate the clinical utility of LLMs, it may be necessary to retrain the model in the clinical context of a medical institution and then evaluate it on a unified benchmark in a large-scale randomized controlled trial.

Seventh, it appears that LLMs are currently not ready for full deployment in clinical settings. While LLMs perform well on various medical licensing exams and may reach or even exceed human capabilities [138-140], this may not suffice for clinical deployment [141]. In the context of clinical practice, LLMs have so far not met the requirements of medical guidelines [87,88,131] for tasks such as medical code extraction and treatment plan development [87,88,142]. They have not reliably interpreted a wide range of medical images [90,143] and have not reached the level of human physicians in clinical diagnosis in various contexts [107,144-146]. In a randomized clinical trial including 50 physicians, the use of LLMs did not significantly improve diagnostic reasoning compared to traditional resources [147]. In addition, there are no MLLMs that can fully handle complex multimodal medical data and efficiently perform most tasks in the clinical workflow. However, recent developments in reasoning LLMs (see the glossary in Multimedia Appendix 1), particularly the emergence of DeepSeek-R1, highlight the potential of applying reinforcement learning to allow models to autonomously explore and generate long chains of thought to solve complex problems [148]. For complex clinical tasks such as differential diagnosis, reasoning LLMs can improve the final clinical decision by leveraging chains of thought to logically analyze a problem step by step [149], self-correcting, and re-evaluating akin to human thinking. Integrating chains of thought and retrieval-enhanced generation has further improved the reasoning ability of the DeepSeek-R1 base model and enhanced the diagnosis of rare diseases [150]. Ongoing research on these models is essential to improve their clinical applicability.

This review has various limitations and potential biases. Without aiming at comprehensively identifying all possible limitations and biases of this review (and the articles included in it), we opt to highlight only key limitations and biases that have a direct impact on the interpretation of the results and may lead to misinterpretation if not well identified and understood. First, we set a citation threshold to select journals, which ineluctably excluded journals below that threshold that may have published important and relevant studies. This may have also led to potential biases if, for example, journals with higher citations were not representative of the literature on clinical applications of LLMs. However, this choice was necessary in our context given the abundant and fast-paced progress in LLMs that makes a systematic and complete evaluation practically impossible. We believe that we incorporated key studies that should hopefully well represent the overall status of the use of LLMs in clinical applications, and we plan to regularly update our interactive guideline, which should hopefully at least partially address these issues. Second, we coined the term *best performance* and associated it with models that performed best in each study from the reviewed literature. It should be mentioned that the level of performance in a context does not guarantee a similar performance in different contexts. Therefore, the frequency of “best performance” of a model should not be interpreted as a metric to compare it with other models but should, instead, highlight that scholars used this model in their research and found that it performed best. The same model may perform differently in the future, in another research context, and with different datasets. It is equally important to note that the use of a specific LLM may be driven by various factors that are independent of its intrinsic performance, such as user interfaces (eg, the language of the application that hosts the LLM). To avoid ambiguity and misinterpretation, we recommend the reader to not interpret this as a performance metric allowing for the identification of the best model but, rather, reflect on how researchers have used and deployed LLMs in clinical applications in their own contexts.

### Conclusions

The rapid rise of LLMs has led to an increase in the number and complexity of available algorithms, programming languages, and IT systems, along with growing technical jargon. The identification of LLMs to carry out specific clinical tasks independently or under the control and supervision of experts represents a growing challenge for the clinical community. This study offered a clinical workflow perspective to identify specific clinical tasks to which different LLMs have been applied. In this review, we classified LLMs by workflow stage and clinical tasks and subtasks. We reported the use frequency, performance, and application details of all identified LLMs and provided the best model use cases for each clinical task. We found that, in some contexts, LLMs may successfully be deployed to assist clinicians in accomplishing diverse clinical tasks. While several noncommercial MLLMs might have accomplished a wide range of clinical tasks, we did not find evidence that generalist clinical LLMs might be successfully applied to a broad spectrum of clinical tasks.

We hope that our review, accompanied by the interactive guideline, will help clinicians select appropriate LLMs for integration into clinical practice, aiming at offering personalized, high-quality, and equitable health care to patients. Future research could consider how the development of generalist clinical LLMs may impact clinical practice and extend this review to domains beyond clinical practice.

## Appendix

Multimedia Appendix 1 The glossary, 5-stage clinical workflow, search strategy tables, and review of reviews.

Multimedia Appendix 2 PRISMA (Preferred Reporting Items for Systematic Reviews and Meta-Analyses) checklist.

## Abbreviations

AGI: artificial general intelligence
AI: artificial intelligence
BERT: bidirectional encoder representations from transformers
CT: computed tomography
ECG: electrocardiography
GPU: graphics processing unit
LLM: large language model
MLLM: multimodal large language model
PRISMA: Preferred Reporting Items for Systematic Reviews and Meta-Analyses

## Data Availability

The datasets generated or analyzed during this study, the codes that generated the figures during this study, and the interactive online guideline are available in the GitHub repository [34].
