# Supplementary material for: Implementing Large Language Models in Health Care: Clinician-Focused Review With Interactive Guideline
Source: J Med Internet Res. 2025 Jul 11;27:e71916. doi: 10.2196/71916 (PMC12299950; doi:10.2196/71916)
Supplement: Multimedia Appendix 1 [file jmir_v27i1e71916_app1.docx]

This is a Multimedia Appendix to a full manuscript published in the J Med Internet Res. For full copyright and citation information see <http://dx.doi.org/10.2196/jmir.xxxx>

# Glossary

**API** Application Programming Interface (API) is a set of predefined rules and protocols for communication and interaction between different software programs [[1](#_ENREF_1)].

**artificial general intelligence** While there is no consensus on what artificial general intelligence (AGI) is, one may view AGI system as a form of artificial intelligence with a general scope with the ability to perform well across various goals and contexts [[2](#_ENREF_2)].

**closed-source LLM** In contrast to [open-source LLM](#_bookmark38), this refers to LLMs whose source code, training data, or model weights are kept confidential by the organization that developed it, and which usually require a license to use. It includes [restricted LLM](#_bookmark42)s as well as commercial LLMs such as ChatGPT .

**computational resources** Hardware and software infrastructure that performs LLM [pre-training](#_bookmark39), [fine-tuning](#_bookmark30), [inference](#_bookmark33), and service typically includes processor (CPU) and graphics card (GPU) performance, memory, storage space, and network capacity [[3](#_ENREF_3)].

**few-shot** The ability of an algorithm to learn new tasks or adapt to new domains by providing only a very small amount of samples (contextual information or examples) without retraining or [fine-tuning](#_bookmark30) [[4](#_ENREF_4)].

**fine-tuning** A machine learning technique that refers to the process of taking a model (e.g., [foundation model](#_bookmark31)) that has been pre-trained on a large data set and further adjusting the model parameters to improve performance by using a task-specific dataset. It is often more efficient than training a model from scratch [[5](#_ENREF_5)].

**foundation model** A class of large-scale algorithms pre-trained on large and diverse datasets that are not limited to a single task but instead, provides a generalized basis for down- stream tasks that can be adapted to specific tasks or domains by [fine-tuning](#_bookmark30) and [in-context](#_bookmark32) [learning](#_bookmark32) [[6](#_ENREF_6)].

**in-context learning** Paradigm that allows an algorithm to learn how to answer a question or perform a task (possibly unseen) based on the specific examples (context) provided to it, without the need for additional model parameter updates or training [[7](#_ENREF_7)].

**inference** The model inference process refers to a trained model applying the knowledge learned during the training phase to new data to generate predictions or accomplish a specific task [[8](#_ENREF_8)].

**MLLM** Multimodal LLMs (MLLM)s refer to LLM-based models with the ability to receive, reason and output multimodal information [[9](#_ENREF_9)] such as text, images and sounds [[10](#_ENREF_10)].

**model size** Number of parameters in an algorithm. In a LLM is the number of learnable parameters such as weights and biases in the neural network [[11](#_ENREF_11)].

**NLP** Natural language process (NLP) is a broad field whose goal is to enable computers to naturally understand and generate human language, becoming a bridge between machine language and human language, thereby achieving natural interaction with human language [[12](#_ENREF_12)].

**open-source LLM** Refers to LLMs where the source code, training data or model weights are freely accessible to the public, allowing for better transparency and customization [[13](#_ENREF_13)].

**pretraining** Unlike task-specific training, the pre-training process refers to a situation where a model learns from large-scale data and encodes knowledge in the absence of a specific task. The encoded knowledge can be further used for training a model for a target task [[14](#_ENREF_14)].

**prompt** A specific instruction (e.g., a paragraph of text or a set of keywords) that describes the desired output or task goal to direct an algorithm to generate a specific type of text or perform a specific type of task [[15](#_ENREF_15)].

**prompt engineering** Practice of designing, refining, and implementing [prompt](#_bookmark40)s or instructions that guide the output of LLMs to help in various tasks [[16](#_ENREF_16)].

**reasoning large language models** LLMs that excel in complex tasks like coding and mathematical proofs, incorporating a ‘thinking’ process tasks that common LLMs struggle with [[17](#_ENREF_17)].

**reinforcement learning** Reinforcement learning from human feedback trains a reward model by collecting human ranking feedback on the model outputs, which can simulate human evaluation and reward of the quality of the generated text. The LLM is then automatically fine-tuned and optimized through iterative algorithms to align the output of the language model with human preferences. This approach may reduce toxic output such text with hateful content and make the output form more human friendly [[18](#_ENREF_18), [19](#_ENREF_19)].

**restricted LLM** LLM introduced in research papers without publicly available code, data, or model weights. Access to restricted LLMs is typically granted only upon request and approval from the original authors or research institutions.

**self-attention** By focusing on all positions in the same sequence to compute the response at a given position in the sequence, the mechanism exhibits a better balance between the ability to model long-term dependencies and computational and statistical efficiency [[20](#_ENREF_20)].

**self-supervised learning** A machine learning algorithm that automatically generates labels from the data itself by predicting certain parts of the data using other parts of the input data [[21](#_ENREF_21)].

**transformer** Deep learning neural network architecture based on [self-attention](#_bookmark43) mechanism, mainly used to process sequence-to-sequence language tasks [[22](#_ENREF_22)].

**zero-shot** The ability of an algorithm to infer and perform unseen tasks by directly applying knowledge learned from [pre-training](#_bookmark39) without any additional samples or performing any additional training or [fine-tuning](#_bookmark30) [[23](#_ENREF_23)].


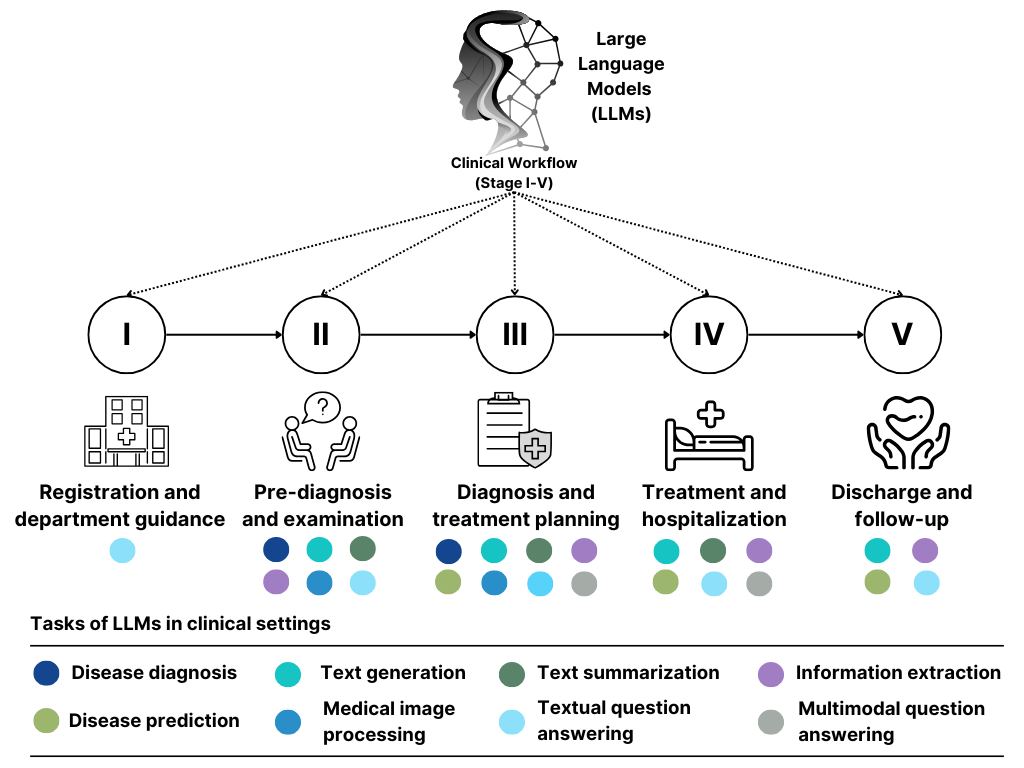


**Figure S1** | **Five-stage clinical workflow.** We considered a patient-oriented clinical workflow, with numbers I-V representing various stages where LLMs may be applied. Stage I: registration and department guidance, Stage II: pre-diagnosis and examination, Stage III: diagnosis and treatment planning, Stage IV: treatment and hospitalization, and Stage V: discharge and follow-up. Clinical tasks of LLMs are represented by colored discs.

**Table S1** | **Literature review: search strategy for the specific keywords in PubMed.** List of search strategy used of academic research work to select relevant work (in PubMed) that applied LLMs to medical (including clinical) studies

| Source | Search strategy |
| --- | --- |
| PubMed | ((GPT[Title/Abstract]) OR (EHR-KnowGen[Title/Abstract]) OR (BARD[Title/Abstract]) OR (PaLM[Title/Abstract]) OR (GatorTron[Title/Abstract]) OR (BERT[Title/Abstract]) OR (Flamingo[Title/Abstract]) OR (Med- MLLM[Title/Abstract]) OR (”Glass AI”[Title/Abstract]) OR (LLaMA[Title/Abstract])) AND ((medic*) OR (clinical) OR (health*)) AND (”large language models”), Timespan: 2022- 01-01 to 2025-03-31, ((ChatRadio-Valuer[Title/Abstract]) OR (PEGASUS[Title/Abstract]) OR (CLIPSyntel[Title/Abstract]) OR (RaDialog[Title/Abstract]) OR (Vicuna[Title/Abstract]) OR (”Bing Chat”[Title/Abstract]) OR (ChatDoc- tor[Title/Abstract]) OR (”Clinical Camel”[Title/Abstract]) OR (Alpaca[Title/Abstract]) OR (MEDITRON[Title/Abstract])) AND ((medic*) OR (clinical) OR (health*)) AND (”large language models”), Timespan: 2022-01-01 to 2025-03-31, ((LLaVA[Title/Abstract]) OR (Claude[Title/Abstract]) OR (NYUTron[Title/Abstract]) OR (T5[Title/Abstract]) OR (EHRTutor[Title/Abstract]) OR (Clinical- BigBird[Title/Abstract]) OR (RadFM[Title/Abstract]) OR (”Multi-modal AI”[Title/Abstract]) OR (PLM- ICD[Title/Abstract]) OR (XLNet[Title/Abstract])) AND ((medic*) OR (clinical) OR (health*)) AND (”large language models”), Timespan: 2022-01-01 to 2025-03-31, ((RoBERTa[Title/Abstract]) OR (BioMega- tron[Title/Abstract]) OR (CAML[Title/Abstract]) OR (TRANS[Title/Abstract]) OR (PromptEHR[Title/Abstract]) OR (MedVINT[Title/Abstract]) OR (SciFive[Title/Abstract]) OR (MOSS[Title/Abstract]) OR (Sensenova[Title/Abstract]) OR (BayLing[Title/Abstract])) AND ((medic*) OR (clinical) OR (health*)) AND (”large language models”), Timespan: 2022-01-01 to 2025-03-31, ((GLM[Title/Abstract]) OR (Bianque[Title/Abstract]) OR (XrayPULSE[Title/Abstract]) OR (BenTsao[Title/Abstract]) OR (Baichuan[Title/Abstract]) OR (Tigerbot[Title/Abstract]) OR (”iFLYTEK Spark”[Title/Abstract]) OR (PGN[Title/Abstract]) OR (BART[Title/Abstract]) OR (OPT[Title/Abstract])) AND ((medic*) OR (clinical) OR (health*)) AND (”large language models”), Timespan: 2022-01-01 to 2025-03-31, ((FLAN- UL2[Title/Abstract]) OR (LongFormer[Title/Abstract]) OR (BLOOMZ[Title/Abstract]) OR (Galactica[Title/Abstract]) OR (Gopher[Title/Abstract]) OR (Chinchilla[Title/Abstract]) OR (DRAGON[Title/Abstract]) OR (Gemini[Title/Abstract]) OR (Mixtral[Title/Abstract]) OR (MPT[Title/Abstract])) AND ((medic*) OR (clinical) OR (health*)) AND (”large language models”), Timespan: 2022-01-01 to 2025-03-31, ((Mistral[Title/Abstract]) OR (Zephyr[Title/Abstract]) OR (Med42[Title/Abstract]) OR (CLIP[Title/Abstract]) OR (M2I2[Title/Abstract]) OR (BioMedLM[Title/Abstract]) OR (Perplexity[Title/Abstract]) OR (Command-xlarge- nightly[Title/Abstract]) OR (BioELECTRa[Title/Abstract]) OR (HeLM[Title/Abstract])) AND ((medic*) OR (clinical) OR (health*)) AND (”large language models”), Timespan: 2022-01-01 to 2024-25-03-31, ((RadioLOGIC[Title/Abstract]) OR (MedChatZH[Title/Abstract]) OR (AlpaCare[Title/Abstract]) OR (MedPromptX[Title/Abstract]) OR (M3D- LaMed[Title/Abstract]) OR (Meerkat[Title/Abstract]) OR (Polaris[Title/Abstract]) OR (MedVersa[Title/Abstract]) OR (MAIRA-1[Title/Abstract]) (DeepSeek[Title/Abstract])) AND ((medic*) OR (clinical) OR (health*)) AND (”large language models”), Timespan: 2022-01-01 to 2025-03-31 |

**Table S2** | **Literature review: search strategy for the specific keywords in ScienceDirect.** List of search strategy of academic research work to select relevant work (in ScienceDirect) that applied LLMs to medical (including clinical) studies.

| Source | Search strategy |
| --- | --- |
| ScienceDirect | ”large language models” Title, abstract, keywords: ((GPT) OR (EHR-KnowGen) OR (BARD) OR (PaLM) OR (GatorTron)) AND ((medical) OR (clinical) OR (health- care) OR (medicine)), Timespan: 2022-01-01 to 2025-03-31, Article type: Research articles; Title, abstract, keywords: ((BERT) OR (Flamingo) OR (Med-MLLM) OR (Glass AI) OR (LLaMA)) AND ((medical) OR (clinical) OR (healthcare) OR (medicine)) , Timespan: 2022-01-01 to 2025-03-31, Article type: Research articles; Title, abstract, keywords: ((LLaVA) OR (Claude) OR (NYUTron) OR (T5) OR (EHRTutor)) AND ((medical) OR (clinical) OR (healthcare) OR (medicine)) , Timespan: 2022-01-01 to 2025-03-31, Article type: Research articles; Title, abstract, keywords: ((”Clinical-BigBird”) OR (RadFM) OR (”Multi- modal AI”) OR (PLM-ICD) OR (XLNet)) AND ((medical) OR (clinical) OR (healthcare) OR (medicine)) , Timespan: 2022-01-01 to 2025-03-31, Article type: Research articles; Title, abstract, keywords: ((ChatRadio-Valuer) OR (PEGASUS) OR (CLIP- Syntel) OR (RaDialog) OR (Vicuna)) AND ((medical) OR (clinical) OR (healthcare) OR (medicine)) , Timespan: 2022-01-01 to 2025-03-31, Article type: Research articles; Title, abstract, keywords: ((”Bing Chat”) OR (ChatDoctor) OR (”Clinical Camel”) OR (Alpaca) OR (MEDITRON)) AND ((medical) OR (clinical) OR (healthcare) OR (medicine)) , Timespan: 2022-01-01 to 2025-03-31, Article type: Research articles; Title, abstract, keywords: ((RoBERTa) OR (BioMegatron) OR (CAML) OR (TRANS) OR (PromptEHR)) AND ((medical) OR (clinical) OR (healthcare) OR (medicine)) , Timespan: 2022-01-01 to 2025-03-31, Article type: Research articles; Title, abstract, keywords: ((MedVINT) OR (SciFive) OR (MOSS) OR (Sensenova) OR (BayLing)) AND ((medical) OR (clinical) OR (healthcare) OR (medicine)) , Timespan: 2022-01-01 to 2025-03-31, Article type: Research articles;Title, abstract, keywords: ((GLM) OR (Bianque) OR (XrayPULSE) OR (BenTsao) OR (Baichuan)) AND ((medical) OR (clinical) OR (healthcare) OR (medicine)) , Timespan: 2022-01-01 to 2025-03-31, Article type: Research articles; Title, abstract, keywords: ((Tigerbot) OR (”iFLYTEK Spark”) OR (PGN) OR (BART) OR (OPT)) AND ((medical) OR (clinical) OR (healthcare) OR (medicine)) , Timespan: 2022-01-01 to 2025-03-31, Article type: Research articles; Title, abstract, keywords: ((FLAN-UL2) OR (LongFormer) OR (BLOOMZ) OR (Galactica) OR (Gopher)) AND ((medical) OR (clinical) OR (healthcare) OR (medicine)) , Timespan: 2022-01-01 to 2025-03-31, Article type: Research articles; Title, abstract, keywords: ((Chinchilla) OR (DRAGON) OR (Gemini) OR (Mixtral) OR (MPT)) AND ((medical) OR (clinical) OR (healthcare) OR (medicine)) , Timespan: 2022-01-01 to 2025-03-31, Article type: Research articles; Title, abstract, keywords: ((Mistral) OR (Zephyr) OR (Med42) OR (CLIP) OR (M2I2)) AND ((medical) OR (clinical) OR (healthcare) OR (medicine)) , Timespan: 2022-01-01 to 2025-03-31, Article type: Research articles; Title, abstract, keywords: ((BioMedLM) OR (Perplexity) OR (Command- xlarge-nightly) OR (BioELECTRa) OR (HeLM)) AND ((medical) OR (clinical) OR (healthcare) OR (medicine)) , Timespan: 2022-01-01 to 2025-03-31, Article type: Research articles; Title, abstract, keywords: ((RadioLOGIC) OR (MedChatZH) OR (AlpaCare) OR (MedPromptX) OR (M3D-LaMed)) AND ((medical) OR (clinical) OR (healthcare) OR (medicine)) , Timespan: 2022-01-01 to 2025-03-31, Article type: Research articles; Title, abstract, keywords: ((Meerkat) OR (Polaris) OR (Med- Versa) OR (MAIRA-1) OR (DeepSeek)) AND ((medical) OR (clinical) OR (healthcare) OR (medicine)) , Timespan: 2022-01-01 to 2025-03-31, Article type: Research articles;. |

**Table S3** | **Literature review: search strategy for the specific keywords in Scopus.** List of search strategy of academic research work to select relevant work (in Scopus) that applied LLMs to medical (including clinical) studies.

| Source | Search strategy |
| --- | --- |
| Scopus | ( TITLE-ABS-KEY ( ”GPT” OR ”EHR-KnowGen” OR ”BARD” OR ”PaLM” OR ”GatorTron” OR ”BERT” OR ”Flamingo” OR ”Med-MLLM” OR ”Glass AI” OR ”LLaMA”) AND TITLE-ABS-KEY ( ”clinical” OR ”medic*” OR ”health*” ) AND ALL(”large language models”) ) AND (LOAD-DATE >20220101) AND (LOAD-DATE <20250331) AND ( LIMIT-TO ( DOCTYPE,”ar” ) ) AND ( LIMIT-TO ( LANGUAGE,”English” ) ), ( TITLE-ABS-KEY ( ”ChatRadio-Valuer” OR ”PEGASUS” OR ”CLIPSyntel” OR ”RaDia- log” OR ”Vicuna” OR ”Bing Chat” OR ”ChatDoctor” OR ”Clinical Camel” OR ”Alpaca” OR ”MEDITRON”) AND TITLE-ABS-KEY ( ”clinical” OR ”medic*” OR ”health*) AND ALL(”large language models”) ) AND (LOAD- DATE >20220101) AND (LOAD-DATE <20250331) AND ( LIMIT-TO ( DOCTYPE,”ar” ) ) AND ( LIMIT-TO ( LANGUAGE,”English” ) ), ( TITLE-ABS-KEY ( ”LLaVA” OR ”Claude” OR ”NYUTron” OR ”T5” OR ”EHRTutor” OR ”Clinical-BigBird” OR ”RadFM” OR ”Multi-modal AI” OR ”PLM-ICD” OR ”XLNet”) AND TITLE-ABS-KEY ( ”clinical” OR ”medic*” OR ”health*” ) AND ALL(”large lan- guage models”) ) AND (LOAD-DATE >20220101) AND (LOAD-DATE <20250331) AND ( LIMIT-TO ( DOC- TYPE,”ar” ) ) AND ( LIMIT-TO ( LANGUAGE,”English”) ), ( TITLE-ABS-KEY ( ”RoBERTa” OR ”BioMegatron” OR ”CAML” OR ”TRANS” OR ”PromptEHR” OR ”MedVINT” OR ”SciFive” OR ”MOSS” OR ”Sensenova” OR ”BayLing”) AND TITLE-ABS-KEY ( ”clinical” OR ”medic*” OR ”health*” ) AND ALL(”large language models”) ) AND (LOAD-DATE >20220101) AND (LOAD-DATE <20250331) AND ( LIMIT-TO ( DOCTYPE,”ar” ) ) AND ( LIMIT- TO ( LANGUAGE,”English” ) ), ( TITLE-ABS-KEY (”GLM” OR ”Bianque” OR ”XrayPULSE” OR ”BenTsao” OR ”Baichuan” OR ”Tigerbot” OR ”iFLYTEK Spark” OR ”PGN” OR ”BART” OR ”OPT”) AND TITLE-ABS-KEY ( ”clinical” OR ”medic*” OR ”health*” ) AND ALL(”large language models”) ) AND (LOAD-DATE >20220101) AND (LOAD-DATE <20250331) AND ( LIMIT-TO ( DOCTYPE,”ar” ) ) AND ( LIMIT-TO ( LANGUAGE,”English” ) ), ( TITLE-ABS-KEY ( ”FLAN-UL2” OR ”LongFormer” OR ”BLOOMZ” OR ”Galactica” OR ”Gopher” OR ”Chin- chilla” OR ”DRAGON” OR ”Gemini” OR ”Mixtral” OR ”MPT”) AND TITLE-ABS-KEY ( ”clinical” OR ”medic*” OR ”health*” ) AND ALL(”large language models”) ) AND (LOAD-DATE >20220101) AND (LOAD-DATE <20250331) AND ( LIMIT-TO ( DOCTYPE,”ar” ) ) AND ( LIMIT-TO ( LANGUAGE,”English” ) ), ( TITLE-ABS-KEY (”Mistral” OR ”Zephyr” OR ”Med42” OR ”CLIP” OR ”M2I2” OR ”BioMedLM” OR ”Perplexity” OR ”Command-xlarge-nightly” OR ”BioELECTRa” OR ”HeLM”) AND TITLE-ABS-KEY ( ”clinical” OR ”medic*” OR ”health*” ) AND ALL(”large language models”) ) AND (LOAD-DATE >20220101) AND (LOAD-DATE <20250331) AND ( LIMIT-TO ( DOCTYPE,”ar” ) ) AND ( LIMIT-TO ( LANGUAGE,”English” ) ), ( TITLE-ABS-KEY ( ”RadioLOGIC OR ”MedChatZH” OR ”AlpaCare” OR ”MedPromptX” OR ”M3D-LaMed” OR ”Meerkat” OR ”Polaris” OR ”MedVersa” OR ”MAIRA-1” OR “DeepSeek”) AND TITLE-ABS-KEY ( ”clinical” OR ”medic*” OR ”health*” ) AND ALL(”large language models”) ) AND (LOAD-DATE >20220101) AND (LOAD-DATE <20250331) AND ( LIMIT-TO ( DOCTYPE,”ar” )) AND ( LIMIT-TO ( LANGUAGE,”English” )) |

\

**Table S4** | **Literature review: search strategy for the specific keywords in arxiv.** List of search strategy of academic research work to select relevant work (in arxiv) that applied LLMs to medical (including clinical) studies.

| Source | Search strategy |
| --- | --- |
| arxiv | Query: order: -announced date first; date range: from 2022-01-01 to 2025-03-31; terms: AND all=”large language models”; AND abstract=medic* OR health* OR clinical; AND abstract=”GPT” OR ”EHR-KnowGen” OR ”BARD” OR ”PaLM” OR ”GatorTron” OR ”BERT” OR ”Flamingo” OR ”Med-MLLM” OR ”Glass AI” OR ”LLaMA”; AND abstract=multimodal, Query: order: -announced date first; date range: from 2022-01-01 to 2025-03-31; terms: AND all=”large language models”; AND abstract=medic* OR health* OR clinical; AND abstract=”ChatRadio-Valuer” OR ”PEGASUS” OR ”CLIPSyntel” OR ”RaDialog” OR ”Vicuna” OR ”Bing Chat” OR ”ChatDoctor” OR ”Clinical Camel” OR ”Alpaca” OR ”MEDITRON” ; AND abstract=multimodal, Query: order: - announced date first; date range: from 2022-01-01 to 2025-03-31; terms: AND all=”large language models”; AND abstract=medic* OR health* OR clinical; AND abstract=”LLaVA” OR ”Claude” OR ”NYUTron” OR ”T5” OR ”EHRTutor” OR ”Clinical-BigBird” OR ”RadFM” OR ”Multi-modal AI” OR ”PLM-ICD” OR ”XLNet” ; AND abstract=multimodal, Query: order: -announced date first; date range: from 2022-01-01 to 2025-03-31; terms: AND all=”large language models”; AND abstract=medic* OR health* OR clin- ical; AND abstract=”RoBERTa” OR ”BioMegatron” OR ”CAML” OR ”TRANS” OR ”PromptEHR” OR ”MedVINT” OR ”SciFive” OR ”MOSS” OR ”Sensenova” OR ”BayLing” ; AND abstract=multimodal, Query: order: -announced date first; date range: from 2022-01-01 to 2025-03-31; terms: AND all=”large language models”; AND abstract=medic* OR health* OR clinical; AND abstract=”GLM” OR ”Bianque” OR ”Xray- PULSE” OR ”BenTsao” OR ”Baichuan” OR ”Tigerbot” OR ”iFLYTEK Spark” OR ”PGN” OR ”BART” OR ”OPT” ; AND abstract=multimodal, Query: order: -announced date first; date range: from 2022-01-01 to 2025-03-31; terms: AND all=”large language models”; AND abstract=medic* OR health* OR clinical; AND abstract=”FLAN-UL2” OR ”LongFormer” OR ”BLOOMZ” OR ”Galactica” OR ”Gopher” OR ”Chinchilla” OR ”DRAGON” OR ”Gemini” OR ”Mixtral” OR ”MPT” ; AND abstract=multimodal, Query: order: -announced date first; date range: from 2022-01-01 to 2025-03-31; terms: AND all=”large language models”; AND abstract=medic* OR health* OR clinical; AND abstract=”Mistral” OR ”Zephyr” OR ”Med42” OR ”CLIP” OR ”M2I2” OR ”BioMedLM” OR ”Perplexity” OR ”Command-xlarge-nightly” OR ”BioELECTRa” OR ”HeLM” ; AND abstract=multimodal, Query: order: -announced date first; date range: from 2022-01-01 to 2025-03-31; terms: AND all=”large language models”; AND abstract=medic* OR health* OR clinical; AND abstract=”RadioLOGIC” OR ”MedChatZH” OR ”AlpaCare” OR ”MedPromptX” OR ”M3D-LaMed” OR ”Meerkat” OR ”Polaris” OR ”MedVersa” OR ”MAIRA-1” OR “DeepSeek” ; AND abstract=multimodal. |

**Table S5** | **Literature review: search strategy for the specific keywords in IEEE Xplore.** List of search strategy of academic research work to select relevant work (in IEEE Xplore) that applied LLMs to medical (including clinical) studies.

| Source | Search strategy |
| --- | --- |
| IEEE Xplore | (((”Abstract”: GPT) OR (”Abstract”: EHR-KnowGen) OR (”Abstract”: BARD) OR (”Abstract”: PaLM) OR (”Ab- stract”: GatorTron) OR (”Abstract”: BERT) OR (”Ab- stract”: Flamingo) OR (”Abstract”: Med-MLLM) OR (”Ab- stract”: ”Glass AI”) OR (”Abstract”: LLaMA) OR (”Ab- stract”: ChatRadio-Valuer) OR (”Abstract”: PEGASUS) OR (”Abstract”: CLIPSyntel) OR (”Abstract”: RaDialog) OR (”Abstract”: Vicuna) OR (”Abstract”: ”Bing Chat”) OR (”Abstract”: ChatDoctor) OR (”Abstract”: ”Clinical Camel”) OR (”Abstract”: Alpaca) OR (”Abstract”: MEDITRON) OR (”Abstract”: LLaVA) OR (”Abstract”: Claude)) AND ((”Abstract”: medic*) OR (”Abstract”: clinical) OR (”Ab- stract”: health*))), ((”Abstract”: NYUTron) OR (”Abstract”: T5) OR (”Abstract”: EHRTutor) OR (”Abstract”: Clinical- BigBird) OR (”Abstract”: RadFM) OR (”Abstract”: ”Multi- modal AI”) OR (”Abstract”: PLM-ICD) OR (”Abstract”: XL- Net) OR (”Abstract”: RoBERTa) OR (”Abstract”: BioMega- tron) OR (”Abstract”: CAML) OR (”Abstract”: TRANS) OR (”Abstract”: PromptEHR) OR (”Abstract”: MedVINT) OR (”Abstract”: SciFive) OR (”Abstract”: MOSS) OR (”Abstract”: Sensenova) OR (”Abstract”: BayLing) OR (”Abstract”: GLM) OR (”Abstract”: Bianque) OR (”Ab- stract”: XrayPULSE) OR (”Abstract”: BenTsao)) AND ((”Abstract”: medic*) OR (”Abstract”: clinical) OR (”Ab- stract”: health*)), (((”Abstract”: Baichuan) OR (”Abstract”: Tigerbot) OR (”Abstract”: ”iFLYTEK Spark”) OR (”Ab- stract”: PGN) OR (”Abstract”: BART) OR (”Abstract”: OPT) OR (”Abstract”: FLAN-UL2) OR (”Abstract”: LongFormer) OR (”Abstract”: BLOOMZ) OR (”Abstract”: Galactica) OR (”Abstract”:Gopher) OR (”Abstract”: Chinchilla) OR (”Ab- stract”: DRAGON) OR (”Abstract”: Gemini) OR (”Abstract”: Mixtral) OR (”Abstract”:MPT) OR (”Abstract”: Mistral) OR (”Abstract”:Zephyr) OR (”Abstract”: Med42) OR (”Abstract”: CLIP) OR (”Abstract”: M2I2) OR (”Abstract”: BioMedLM)) AND ((”Abstract”: medic*) OR (”Abstract”: clinical) OR (”Abstract”: health*))), (((”Abstract”: Perplexity) OR (”Ab- stract”: Command-xlarge-nightly) OR (”Abstract”: BioELEC- TRa) OR (”Abstract”: HeLM) OR (”Abstract”: RadioLOGIC) OR (”Abstract”: MedChatZH) OR (”Abstract”: AlpaCare) OR (”Abstract”: MedPromptX) OR (”Abstract”: M3D-LaMed) OR (”Abstract”: Meerkat) OR (”Abstract”:Polaris) OR (”Ab- stract”: MedVersa) OR (”Abstract”: MAIRA-1) OR (”Abstract”: DeepSeek)) AND ((”Ab- stract”: medic*) OR (”Abstract”: clinical) OR (”Abstract”: health*))). |

To clarify the unique contributions, strengths, and limitations of our study, we identified the 30 most cited review articles on the application of LLMs in clinical medicine published between 2023 and 2025 (10 articles per year). We systematically compared these reviews based on three consistent criteria: 1. model scope 2. review scope 3. review focus. Our findings show that while most of the reviews discussed AI or LLM applications broadly, and some focused specifically on clinical applications of ChatGPT, only one review entitled "The breakthrough of large language models release for medical applications: 1-year timeline and perspectives" provides a timeline of milestone models such as ChatGPT and Med-PaLM. In contrast, our review specifically summarized 330 LLMs extracted from all articles that met the inclusion criteria from January 1, 2022 to March 31, 2025, and categorized them according to clinical task, subtask category, and application stage in a five-stage clinical workflow. In addition, our study additionally provided an interactive online guidance tool specifically designed to guide clinicians in selecting appropriate LLMs for specific clinical tasks. This systematic comparison highlights the clinical relevance and practical usability of our study.

**Table S6** | **Review of reviews:** summary of the top 30 most highly cited review articles published between 2023 and 2025 (10 per year) on the clinical application of large language models. The table compares the model scope, review scope, and review focus for each review.

| **Authors (year)** | **Title** | **Model scope** | **Review scope** | **Review focus** |
| --- | --- | --- | --- | --- |
| Thirunavukarasu et al. [[24](#_ENREF_24)]  (2023) | Large language models in medicine | LLMs | Medical applications | The background, models, medical applications, challenges and future prospects of LLMs in the medical field |
| Liu et al. [[25](#_ENREF_25)]  (2023) | Summary of chatgpt-related research and perspective towards the future of large language models | ChatGPT | Applications in different fields (education, medicine, others) | ChatGPT related work includes applications and ethics and limitations |
| Dave et al. [[26](#_ENREF_26)]  (2023) | ChatGPT in medicine: an overview of its applications, advantages, limitations, future prospects, and ethical considerations | ChatGPT | Medical applications | Advantages, limitations, ethical considerations, future prospects, and practical applications of ChatGPT and artificial intelligence (AI) in healthcare and medicine |
| Hadi et al. [[27](#_ENREF_27)]  (2023) | A survey on large language models: Applications, challenges, limitations, and practical usage | LLMs | Comprehensive overview of LLMs | History, architecture, training methods, applications, and challenges of LLMs |
| Rawte et al. [[28](#_ENREF_28)]  (2023) | A survey of hallucination in large foundation models | Foundation models | Hallucination | Classification, evaluation criteria, mitigation strategies and future development of hallucination phenomena in large foundation models |
| Malik Sallam [[29](#_ENREF_29)]  (2023) | The utility of ChatGPT as an example of large language models in healthcare education, research and practice: Systematic review on the future perspectives and Potential Limitations | ChatGPT | Healthcare education, research and practice | Identify future prospects and potential limitations of ChatGPT in healthcare education, academic/scientific writing, and healthcare practice |
| Rajpurkar et al. [[30](#_ENREF_30)]  (2023) | The current and future state of AI interpretation of medical images | AI models | Radiological applications | Advances, challenges, and opportunities in the development of AI models for radiology and their application in clinical practice. |
| Huang et al. [[31](#_ENREF_31)]  (2023) | ChatGPT for shaping the future of dentistry: the potential of multi-modal large language model | Multimodal LLMs | Dental applications | Deployment methods of LLMs in dental diagnostics, and the potential of fully automatic multimodal LLM artificial intelligence systems in dental clinical applications. |
| Zhang et al. [[32](#_ENREF_32)]  (2023) | Generative AI in medicine and healthcare: promises, opportunities and challenges | Generative AI | Medical applications | Applications and related issues of generative AI in medicine and healthcare |
| Yang et al. [[33](#_ENREF_33)]  (2023) | Large language models in health care: Development, applications, and challenges | LLMs | Biomedical or clinical applications | The development of LLMs designed for biomedical or clinical applications, and their potential applications and trial applications and challenges in clinical Settings |
| Chang et al. [[34](#_ENREF_34)]  (2024) | A survey on evaluation of large language models | LLMs | Evaluation methods | An overview of evaluation tasks, encapsulating evaluation tasks, protocols and benchmarks, and future challenges |
| Raiaan et al. [[35](#_ENREF_35)]  (2024) | A review on large language models: Architectures, applications, taxonomies, open issues and challenges | LLMs | Comprehensive overview of LLMs | An overview of LLMs, including the history, architecture, changes, resources, training methods, applications, impacts, and challenges |
| Tian et al. [[36](#_ENREF_36)]  (2024) | Opportunities and challenges for ChatGPT and large language models in biomedicine and health | LLMs | Biomedical and health applications | Applications and challenges of large language models in biomedicine and health |
| Omiye et al. [[37](#_ENREF_37)]  (2024) | Large language models in medicine: the potentials and pitfalls: a narrative review | LLMs | Medical applications | Introduction, development, applications, and limitations of LLMs |
| Nazi et al. [[38](#_ENREF_38)]  (2024) | Large language models in healthcare and medical domain: A review | LLMs | Medical applications | Applications and challenges of large language models in healthcare. As well as the comparison of state-of-the-art LLMs, the use of open source LLMs and the evaluation indicators of LLMs in the biomedical field |
| Rajesh Bhayana [[39](#_ENREF_39)]  (2024) | Chatbots and large language models in radiology: a practical primer for clinical and research applications | LLMs | Radiological applications | Limitations of LLMs and mitigation strategies as well as applications and future developments in radiology |
| Ullah et al. [[40](#_ENREF_40)]  (2024) | Challenges and barriers of using large language models (LLM) such as ChatGPT for diagnostic medicine with a focus on digital pathology–a recent scoping review | LLMs | Diagnostic medical applications | Several challenges and obstacles associated with the use of large language models in diagnostic medicine |
| Cascella et al. [[41](#_ENREF_41)]  (2024) | The breakthrough of large language models release for medical applications: 1-year timeline and perspectives | LLMs in 2023 | Medical applications | Provides an overview of 2023's large language models, the potential uses and application prospects in the medical field |
| D’Antonoli et al. [[42](#_ENREF_42)]  (2024) | Large language models in radiology: fundamentals, applications, ethical considerations, risks, and future directions | LLMs | Radiological applications | History of LLMs, technology, ChatGPT, rapid engineering, potential applications in medicine and radiology, advantages, disadvantages, and risks, ethical and regulatory considerations, and future directions. |
| Haltaufderheide et al. [[43](#_ENREF_43)]  (2024) | The ethics of ChatGPT in medicine and healthcare: a systematic review on Large Language Models (LLMs) | LLMs | Ethical landscape of medical applications | Describe the ethically relevant applications of LLMs in medicine and healthcare, the main outcomes, and the opportunities, risks, benefits, and potential harms |
| Das et al. [[44](#_ENREF_44)]  (2025) | Security and privacy challenges of large language models: A survey | LLMs | Security and privacy risks | The extent of large language model vulnerabilities, emerging large language model security and privacy attacks, and potential defense mechanisms. |
| Zhang et al. [[45](#_ENREF_45)]  (2025) | Artificial intelligence in drug development | AI | Applications in drug development | An overview of AI applications throughout the drug development process, current challenges and future research directions. |
| Khan et al. [[46](#_ENREF_46)]  2025 | A comprehensive survey of foundation models in medicine | Foundation models | Medical applications | History, learning strategies, flagship models, application classifications, and challenges of foundation models. |
| Strika et al. [[47](#_ENREF_47)]  (2025) | Bridging healthcare gaps: a scoping review on the role of artificial intelligence, deep learning, and large language models in alleviating problems in medical deserts | LLMs | Integration of LLMs with e-health and the Internet of Medical Things | Examines how AI, specifically LLMs, can address the "medical desert" challenge by combining it with e-health and the Internet of Medical Things. |
| Ng et al. [[48](#_ENREF_48)]  (2025) | RAG in health care: a novel framework for improving communication and decision-making by addressing LLM limitations | LLMs | Retrieval-augmented generation methods | Current and future use cases of RAG framework for healthcare information exchange in clinical and industrial settings. |
| Omar et al. [[49](#_ENREF_49)]  (2025) | Evaluating and addressing demographic disparities in medical large language models: a systematic review | LLMs | Demographic bias | Types of demographic bias in LLMs, assessment measures, and mitigation strategies. |
| Farhadi et al. [[50](#_ENREF_50)]  (2025) | Transforming dental diagnostics with artificial intelligence: advanced integration of ChatGPT and large language models for patient care | LLMs (ChatGPT) | Dental applications | The application status, challenges and future development of large language models in the field of oral surgery |
| Busch et al. [[51](#_ENREF_51)]  (2025) | Current applications and challenges in large language models for patient care: a systematic review | LLMs | Applications in patient care | Application and limitations of LLMs in patient care |
| Shiwlani et al. [[52](#_ENREF_52)]  (2025) | Leveraging Generative AI for Precision Medicine: Interpreting Immune Biomarker Data from EHRs in Autoimmune and Infectious Diseases | Generative AI | Applications in precision medicine | Applications and challenges of generative artificial intelligence in the interpretation of immune biomarkers in precision medicine |
| Huo et al. [[53](#_ENREF_53)]  (2025) | Large Language Models for Chatbot Health Advice Studies: A Systematic Review | LLMs (Chatbots) | Clinical integration of chatbots | Chatbot selection, query methods, and performance evaluation in healthcare |

**Reference**

1. Ofoeda J, Boateng R, Effah J. Application programming interface (API) research: A review of the past to inform the future. International Journal of Enterprise Information Systems (IJEIS). 2019 2019;15(3):76–95.

2. Goertzel B. Artificial general intelligence: concept, state of the art, and future prospects. Journal of Artificial General Intelligence. 2014 2014;5(1):1.

3. Strubell E, Ganesh A, McCallum A. Energy and policy considerations for deep learning in NLP. Preprint at arXiv <https://doiorg/1048550/arXiv190602243>. 2019 2019.

4. Wang Y, Yao Q, Kwok JT, Ni LM. Generalizing from a few examples: A survey on few-shot learning. ACM computing surveys (csur). 2020 2020;53(3):1–34.

5. Tajbakhsh N, Shin JY, Gurudu SR, Hurst RT, Kendall CB, Gotway MB, et al. Convolutional neural networks for medical image analysis: Full training or fine tuning? IEEE transactions on medical imaging. 2016 2016;35(5):1299–312.

6. Moor M, Banerjee O, Abad ZSH, Krumholz HM, Leskovec J, Topol EJ, et al. Foundation models for generalist medical artificial intelligence. Nature. 2023 2023;616(7956):259–65.

7. Dong Q, Li L, Dai D, Zheng C, Wu Z, Chang B, et al. A survey on in-context learning. Preprint at arXiv <https://doiorg/1048550/arXiv230100234>. 2022 2022.

8. Wetzstein G, Ozcan A, Gigan S, Fan S, Englund D, Soljačić M, et al. Inference in artificial intelligence with deep optics and photonics. Nature. 2020 2020;588(7836):39–47.

9. Zhang D, Yu Y, Li C, Dong J, Su D, Chu C, et al. Mm-llms: Recent advances in multimodal large language models. arXiv preprint arXiv:240113601. 2024 2024.

10. Yin S, Fu C, Zhao S, Li K, Sun X, Xu T, et al. A survey on multimodal large language models. Preprint at arXiv <https://doiorg/1048550/arXiv230613549>. 2023 2023.

11. Hu X, Chu L, Pei J, Liu W, Bian J. Model complexity of deep learning: A survey. Knowledge and Information Systems. 2021 2021;63:2585–619.

12. Harrer S. Attention is not all you need: the complicated case of ethically using large language models in healthcare and medicine. EBioMedicine. 2023 2023;90.

13. Zhang G, Jin Q, Zhou Y, Wang S, Idnay B, Luo Y, et al. Closing the gap between open source and commercial large language models for medical evidence summarization. NPJ Digital Medicine. 2024 2024;7(1):239.

14. Han X, Zhang Z, Ding N, Gu Y, Liu X, Huo Y, et al. Pre-trained models: Past, present and future. AI Open. 2021 2021;2:225–50.

15. Jiang Z, Xu FF, Araki J, Neubig G. How can we know what language models know? Transactions of the Association for Computational Linguistics. 2020 2020;8:423–38.

16. Meskó B. Prompt engineering as an important emerging skill for medical professionals: tutorial. Journal of medical Internet research. 2023;25:e50638.

17. Li Z-Z, Zhang D, Zhang M-L, Zhang J, Liu Z, Yao Y, et al. From system 1 to system 2: A survey of reasoning large language models. arXiv preprint arXiv:250217419. 2025.

18. Ouyang L, Wu J, Jiang X, Almeida D, Wainwright C, Mishkin P, et al. Training language models to follow instructions with human feedback. Advances in Neural Information Processing Systems. 2022 2022;35:27730–44.

19. Bai Y, Jones A, Ndousse K, Askell A, Chen A, DasSarma N, et al. Training a helpful and harmless assistant with reinforcement learning from human feedback. arXiv preprint arXiv:220405862. 2022 2022.

20. Zhang H, Goodfellow I, Metaxas D, Odena A, editors. Self-attention generative adversarial networks. 2019 2019: PMLR.

21. Liu X, Zhang F, Hou Z, Mian L, Wang Z, Zhang J, et al. Self-supervised learning: Generative or contrastive. IEEE transactions on knowledge and data engineering. 2021;35(1):857-76.

22. Vaswani A, Shazeer N, Parmar N, Uszkoreit J, Jones L, Gomez AN, et al. Attention is all you need. Advances in neural information processing systems. 2017 2017;30.

23. Xian Y, Lampert CH, Schiele B, Akata Z. Zero-shot learning—a comprehensive evaluation of the good, the bad and the ugly. IEEE transactions on pattern analysis and machine intelligence. 2018 2018;41(9):2251–65.

24. Thirunavukarasu AJ, Ting DSJ, Elangovan K, Gutierrez L, Tan TF, Ting DSW. Large language models in medicine. Nature medicine. 2023 2023;29(8):1930–40.

25. Liu Y, Han T, Ma S, Zhang J, Yang Y, Tian J, et al. Summary of chatgpt-related research and perspective towards the future of large language models. Meta-radiology. 2023;1(2):100017.

26. Dave T, Athaluri SA, Singh S. ChatGPT in medicine: an overview of its applications, advantages, limitations, future prospects, and ethical considerations. Frontiers in artificial intelligence. 2023;6:1169595.

27. Hadi MU, Qureshi R, Shah A, Irfan M, Zafar A, Shaikh MB, et al. A survey on large language models: Applications, challenges, limitations, and practical usage. Authorea Preprints. 2023.

28. Rawte V, Sheth A, Das A. A survey of hallucination in large foundation models. arXiv preprint arXiv:230905922. 2023.

29. Sallam M. The utility of ChatGPT as an example of large language models in healthcare education, research and practice: Systematic review on the future perspectives and potential limitations. MedRxiv. 2023:2023.02. 19.23286155.

30. Rajpurkar P, Lungren MP. The current and future state of AI interpretation of medical images. New England Journal of Medicine. 2023;388(21):1981-90.

31. Huang H, Zheng O, Wang D, Yin J, Wang Z, Ding S, et al. ChatGPT for shaping the future of dentistry: the potential of multi-modal large language model. International Journal of Oral Science. 2023;15(1):29.

32. Zhang P, Kamel Boulos MN. Generative AI in medicine and healthcare: promises, opportunities and challenges. Future Internet. 2023;15(9):286.

33. Yang R, Tan TF, Lu W, Thirunavukarasu AJ, Ting DSW, Liu N. Large language models in health care: Development, applications, and challenges. Health Care Science. 2023;2(4):255-63.

34. Chang Y, Wang X, Wang J, Wu Y, Yang L, Zhu K, et al. A survey on evaluation of large language models. ACM transactions on intelligent systems and technology. 2024;15(3):1-45.

35. Raiaan MAK, Mukta MSH, Fatema K, Fahad NM, Sakib S, Mim MMJ, et al. A review on large language models: Architectures, applications, taxonomies, open issues and challenges. IEEE access. 2024;12:26839-74.

36. Tian S, Jin Q, Yeganova L, Lai P-T, Zhu Q, Chen X, et al. Opportunities and challenges for ChatGPT and large language models in biomedicine and health. Briefings in Bioinformatics. 2024;25(1):bbad493.

37. Omiye JA, Gui H, Rezaei SJ, Zou J, Daneshjou R. Large language models in medicine: the potentials and pitfalls: a narrative review. Annals of internal medicine. 2024;177(2):210-20.

38. Nazi ZA, Peng W, editors. Large language models in healthcare and medical domain: A review. Informatics; 2024: MDPI.

39. Bhayana R. Chatbots and large language models in radiology: a practical primer for clinical and research applications. Radiology. 2024;310(1):e232756.

40. Ullah E, Parwani A, Baig MM, Singh R. Challenges and barriers of using large language models (LLM) such as ChatGPT for diagnostic medicine with a focus on digital pathology–a recent scoping review. Diagnostic pathology. 2024;19(1):43.

41. Cascella M, Semeraro F, Montomoli J, Bellini V, Piazza O, Bignami E. The breakthrough of large language models release for medical applications: 1-year timeline and perspectives. Journal of Medical Systems. 2024;48(1):22.

42. D’Antonoli TA, Stanzione A, Bluethgen C, Vernuccio F, Ugga L, Klontzas ME, et al. Large language models in radiology: fundamentals, applications, ethical considerations, risks, and future directions. Diagnostic and Interventional Radiology. 2024;30(2):80.

43. Haltaufderheide J, Ranisch R. The ethics of ChatGPT in medicine and healthcare: a systematic review on Large Language Models (LLMs). NPJ digital medicine. 2024;7(1):183.

44. Das BC, Amini MH, Wu Y. Security and privacy challenges of large language models: A survey. ACM Computing Surveys. 2025;57(6):1-39.

45. Zhang K, Yang X, Wang Y, Yu Y, Huang N, Li G, et al. Artificial intelligence in drug development. Nature Medicine. 2025:1-15.

46. Khan W, Leem S, See KB, Wong JK, Zhang S, Fang R. A comprehensive survey of foundation models in medicine. IEEE Reviews in Biomedical Engineering. 2025.

47. Strika Z, Petkovic K, Likic R, Batenburg R. Bridging healthcare gaps: a scoping review on the role of artificial intelligence, deep learning, and large language models in alleviating problems in medical deserts. Postgraduate medical journal. 2025;101(1191):4-16.

48. Ng KKY, Matsuba I, Zhang PC. RAG in health care: a novel framework for improving communication and decision-making by addressing LLM limitations. NEJM AI. 2025;2(1):AIra2400380.

49. Omar M, Sorin V, Agbareia R, Apakama DU, Soroush A, Sakhuja A, et al. Evaluating and addressing demographic disparities in medical large language models: a systematic review. International Journal for Equity in Health. 2025;24(1):57.

50. Farhadi Nia M, Ahmadi M, Irankhah E. Transforming dental diagnostics with artificial intelligence: advanced integration of ChatGPT and large language models for patient care. Frontiers in Dental Medicine. 2025;5:1456208.

51. Busch F, Hoffmann L, Rueger C, van Dijk EH, Kader R, Ortiz-Prado E, et al. Current applications and challenges in large language models for patient care: a systematic review. Communications Medicine. 2025;5(1):26.

52. Shiwlani A, Kumar S, Qureshi HA. Leveraging Generative AI for Precision Medicine: Interpreting Immune Biomarker Data from EHRs in Autoimmune and Infectious Diseases. Annals of Human and Social Sciences. 2025;6(1):244-60.

53. Huo B, Boyle A, Marfo N, Tangamornsuksan W, Steen JP, McKechnie T, et al. Large Language Models for Chatbot Health Advice Studies: A Systematic Review. JAMA Network Open. 2025;8(2):e2457879-e.
